# A mechanistic view on lodging resistance in rye and wheat: a multiscale comparative study

**DOI:** 10.1111/pbi.13689

**Published:** 2021-09-12

**Authors:** Aleksandra Muszynska, Andre Guendel, Michael Melzer, Yudelsy Antonia Tandron Moya, Marion S. Röder, Hardy Rolletschek, Twan Rutten, Eberhard Munz, Gilbert Melz, Stefan Ortleb, Ljudmilla Borisjuk, Andreas Börner

**Affiliations:** ^1^ Leibniz Institute of Plant Genetics and Crop Plant Research (IPK) Stadt Seeland Germany; ^2^ Institute of Experimental Physics 5 University of Würzburg Würzburg Germany; ^3^ Nordic Seed Germany GmbH Nienstädt Germany

**Keywords:** cell wall, scanning electron microscopy, Fourier‐transform infrared spectroscopy, lodging resistance, nuclear magnetic resonance imaging, QTL mapping

## Abstract

The development of crop varieties that are resistant to lodging is a top priority for breeding programmes. Herein, we characterize the rye mutant ´Stabilstroh’ (‘stable straw’) possessing an exceptional combination of high lodging resistance, tall posture and high biomass production. Nuclear magnetic resonance imaging displayed the 3‐dimensional assembly of vascular bundles in stem. A higher number of vascular bundles and a higher degree of their incline were the features of lodging‐resistant versus lodging‐prone lines. Histology and electron microscopy revealed that stems are fortified by a higher proportion of sclerenchyma and thickened cell walls, as well as some epidermal invaginations. Biochemical analysis using Fourier‐transform infrared spectroscopy and inductively coupled plasma‐optical emission spectrometry further identified elevated levels of lignin, xylan, zinc and silicon as features associated with high lodging resistance. Combined effects of above features caused superior culm stability. A simplistic mathematical model showed how mechanical forces distribute within the stem under stress. Main traits of the lodging‐resistant parental line were heritable and could be traced back to the genetic structure of the mutant. Evaluation of lodging‐resistant wheat ‘Babax’ (‘Baviacora’) versus contrasting, lodging‐prone, genotype ´Pastor´ agreed with above findings on rye. Our findings on mechanical stability and extraordinary culm properties may be important for breeders for the improvement of lodging resistance of tall posture cereal crops.

## Introduction

Lodging is the phenomenon of small‐grained cereal shoot displacement from the vertical stance. This occurs after ear or panicle emergence, and results in breakage or bending of the stems, making the shoots lean towards the ground. The resulting low grain quality and quantity (up to 80% yield reduction) increased drying costs, and harvesting issues lead to significant economic losses (Atkins, 1938; Berry, 2019; Kelbert *et al*., 2004; Kong *et al*., 2013). Since lodging is usually associated with fungal infections and mycotoxin contamination, it also poses a potential health risk (Berry *et al*., 2003, 2004; Pinthus, 1973).

Lodging can be classified into two types: stem lodging, caused by buckling or breaking of the basal stem internodes (Berry *et al*., 2004; Pinthus, 1973); and root lodging, caused by the failure of efficient root anchorage (Berry *et al*., 2004; Crook and Ennos, 1993; Mulder, 1954; Pinthus, 1973). This effect is influenced by environmental factors, in particular rain and wind, which have led to more extreme events occurring in the last few decades due to the climate change (Liu *et al*., 2019; Otto *et al*., 2020; Young and Ribal, 2019). Lodging occurs particularly under growth‐promoting conditions, since fast growth affects the length of the basal internodes and weakens the base of the stem (Berry *et al*., 2004; Chalmers *et al*., 1998; Crook and Ennos, 1995).

The first successful attempt against lodging was achieved during the 1950s and ‘60s with the development of shorter and stiffer straw by introduction of dwarf and semi‐dwarf varieties. In more recent decades, dwarfing genes have served as useful tools for combatting this phenomenon (Berry *et al*., 2004; Fischer and Stapper, 1987). However, the use of these genes has not eliminated lodging and has not always resulted in positive pleiotropic effects on grain yield, as observed for certain wheat and rye dwarfing genes (Miedaner *et al*., 2011; Milach and Federizzi, 2001). In addition, the minimum plant height for optimum grain yield of approximately 0.7 m has already been reached in certain genotypes (Flintham *et al*., 1997), making it unlikely that further resistance to lodging would be achieved by further shortening the tillers.

Great potential lies in the ability to increase the stem base strength and flexural rigidity, and therefore, in uncovering the genetic regulation of these traits for the purpose of aiding the selection process of plant breeders. Characteristics affecting the mechanical properties of culms include the sclerenchymal tissue thickness (Kong *et al*., 2013), the number of vascular bundles (Packa *et al*., 2015; Wang *et al*., 2006) and the deposition of cellulose and lignin in the secondary cell wall (CW) (Jones *et al*., 2001; Kong *et al*., 2013). The chemical basis of structurally rigid formations within the shoot is a subject of much debate. Certain lignin monomers and the variation between amorphous and crystalline sub‐elements in structural (like holocelluloses) and non‐structural (like sucrose) carbohydrates pools (Muhammad *et al*., 2020; Zhang *et al*., 2020) are of particular interest in the context of lodging resistance (Luo *et al*., 2019; Muhammad *et al*., 2020). The capacity of carbohydrates to hold water has a positive effect on plant stability during growth (Kölln, 2004; Lee *et al*., 2020). Vascular bundles and surrounding leaf sheaths are also significant contributors to lodging resistance (Lee *et al*., 2020; Wójtowicz *et al*., 2020; Wu and Ma, 2020; Zhou *et al*., 2019). Overall, it can be concluded that linking chemical to architectural/anatomical traits is the most promising approach to understand lodging resistance in cereal crops (von Forell *et al*., 2015).

In our present study, a multidisciplinary approach was applied to provide a mechanistic view on lodging resistance in rye and wheat. For this purpose, we investigated the lodging‐resistant rye mutant ‘Stabilstroh’ (Ger. ‘Stable Straw’) and the lodging‐resistant wheat lines ‘Babax’ (’Baviacora’) (Pinto and Reynolds, 2015; Tripathi *et al*., 2003) in comparison with respective lodging‐prone cultivars. The spontaneous ‘Stabilstroh’ mutant exhibits a striking phenotype characterized by high lodging resistance combined with long culms. This line was earlier used to develop two hybrid cultivars characterized by remarkable resistance to lodging and high biomass production (Beschreibende Sortenliste, 2009). Here, we identify biochemical, morphological and anatomical traits characteristic for high lodging resistance. Based on features identified by nuclear magnetic resonance imaging (MRI) and the stem´s biochemical composition, we constructed a simplistic model to evaluate the mechanical resistance of stems. The rye mapping population was further used to study underlying QTLs.

## Results

### The rye ‘Stabilstroh’ genotype leads to a distinct culm architecture

**Figure 1:**
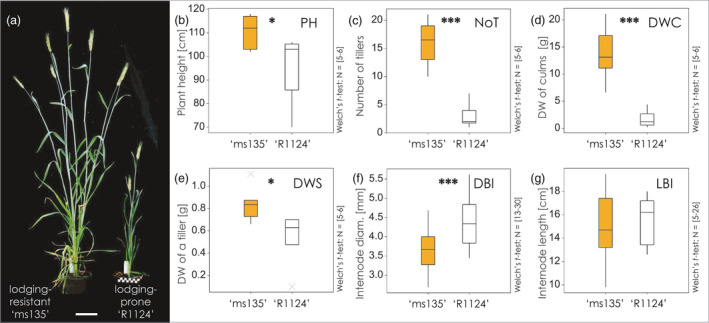
Comparative analysis of morphology and anatomy of lodging‐resistant (‘ms135’) and lodging‐prone (‘R1124’) line of rye. (a) Plant phenotype, (b) Plant height (PH), (c) Number of tillers (NoT), (d) Dry weight of culms (DWC), (e) Dry weight of a single tiller (DWS), (f) Diameter of the second basal Internode (DBI), (g) Length of the second basal internode (LBI). Data are shown as boxplots with median values with whiskers indicating variability outside the upper and lower quartiles in the two lines (‘ms135’ and ‘R1124’). Bar = 10 cm. Welch’s *t*‐test; *P < 0.05; **P < 0.01; ***P < 0.001.

The lodging‐resistant ‘Stabilstroh’ plants (‘ms135’ line) were taller with more extensive tillering than the lodging‐prone plants (‘R1124’ line) (Figure 1a–c). A 15 cm difference in average plant height (PH) was observed between the plant types (110.7 ± 6.9 cm for ‘ms135’ vs. 95 ± 15.2 cm for ‘R1124’). The lodging‐resistant line generated ˜5‐fold higher number of tillers (NoT) per plant compared with the lodging‐prone line. The dry weight of the culms (DWC) was eightfold higher in the resistant line and reached 13.7 ± 5.0 g, compared with only 1.7 ± 1.6 g in the ‘R1124’ plants (Figure 1d). There were notable differences in the diameter of the basal internodes (DBI), with those in mutant line being unexpectedly smaller than those in the ‘R1124’ line (3.6 ± 0.5 mm vs. 4.4 ± 0.6 mm) (Figure 1f). The length of the second basal internode (LBI) was similar between the two lines (Figure 1g). We recorded the morphological parameters (PH, DBI, LBI, NoT and DWC) in 129 plants of the 304/1 F_2_ segregating population and compared them to those of the parental lines (external morphology, Table S1). The key features in the progeny were normally distributed, and certain traits, such as PH and DW, were more similar to those of the lodging‐resistant parent, implying additive inheritance with a small dominance effect. Overall, the lodging‐resistant plants were taller, produced more tillers and developed a heavier, but thinner, culm than the lodging‐prone plants.

### Lodging‐resistant rye plants exhibit different vasculature arrangement

**Figure 2:**
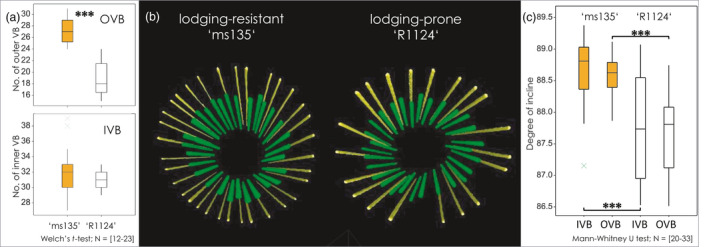
The arrangement of vascular bundles in lodging‐resistant (‘ms135’) and lodging‐prone line (‘R1124’). (a) Number of outer and inner vascular bundles (OVB; IVB). (b) Model of individual vascular bundles in inner (green) and outer (yellow) vascular ring by MRI‐based 3D modelling. (c) Degree of the incline of vascular bundles. Welch’s *t*‐test (a)/Mann–Whitney (c); *P < 0.05; **P < 0.01; ***P < 0.001.

The internodes contained two vascular rings consisting of numerous vascular bundles (Figure 2b). The inner veins supply the next leaf, the outer veins will first fuse before supplying the leaf after that. By combined use of light microscopy (LM) and MRI‐modelling, we demonstrated that the number of outer vascular bundles (OVB) was significantly higher (*P* < 0.0001) in the lodging‐resistant plants compared with the lodging‐prone ones (27.4 ± 2.1 vs. 18.8 ± 2.8, respectively) (Figure 2a). The number of inner vascular bundles (IVB) was comparable in the two lines. To understand the relevance of this in the resistance to lodging, we visualized the spatial arrangement of the vascular bundles using a non‐invasive MRI approach (Figure 2b). The resulting 3D model visualized the different arrangement of the vasculature, displaying a higher degree of incline of the vascular bundles in the resistant plants (Figure 2c). Thus, specific features of the stem in lodging‐resistant plants appear to be the spatial arrangement and larger numbers of vascular bundles within the outer ring. In combination with the thinner culm, the most striking difference seems the per area number of bundles.

### Culms of lodging‐resistant rye plants have distinct cellular features

**Figure 3:**
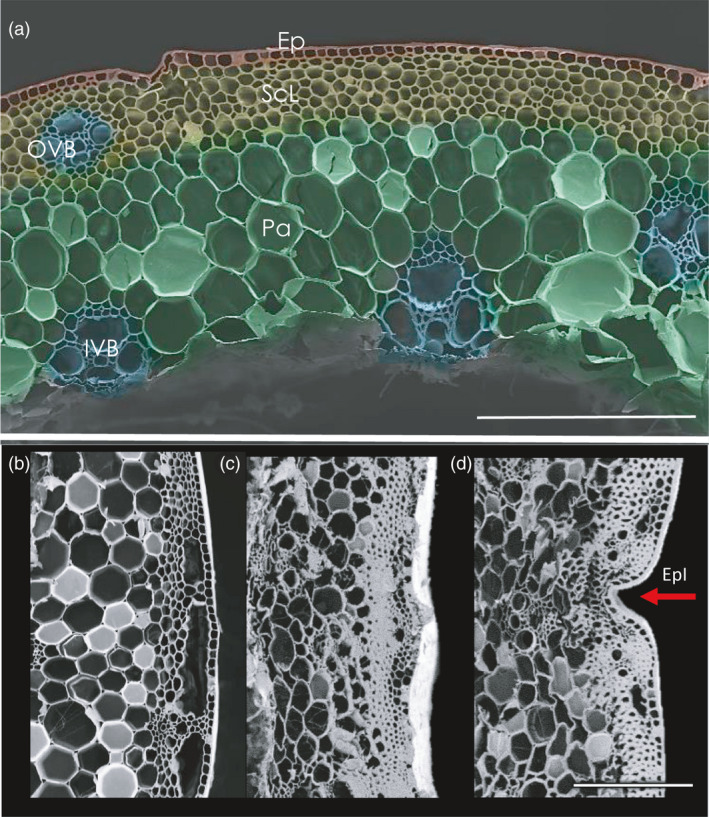
Scanning electron microscopic analysis of stem anatomy of lodging‐resistant (‘ms135’) and lodging‐prone (‘R1124’) line. (a) SEM image of stem in line ‘R1124’, (b) EM image showing smooth surface of stem, (c,d) Characteristic alteration of the surface in the resistant line, where (c) shows wavy surface and (d) the epidermal invagination (EpI; red arrowhead). Data shown are for the second basal internode. Abbreviations: CWT – culm wall thickness, Ep – epidermis, ScL –sclerenchyma tissue layer, Pa – parenchyma, IVB – inner vascular bundle, OVB – outer vascular bundle; bars = 200 μm.

A comparative anatomical and morphological analysis of internodes of resistant and prone plants was carried out by LM and scanning electron microscopy (SEM) and the following structural key traits could be determined as follows: a single epidermal layer, 5–6 rows of sclerenchyma cells with a series of outer and inner vascular bundles embedded in several layers of parenchymal cells (Figure 3a). The wrinkled surface of the culm in the lodging‐resistant line plants was characterized by epidermal invaginations (EpI), as observed by SEM (Figure 3b,c). The culm walls of the lodging‐prone plants appeared less‐condensed and exhibited a smoother surface (Figure 3d). The sclerenchymal tissue layer (ScL) in the ‘ms135’ line was 70.8 ± 13.1 μm thick, whereas it was only 49.6 ± 10.2 μm thick in the ‘R1124’ line (Table S1). The OVBs were embedded in the ScL (Figure S3).

**Figure 4:**
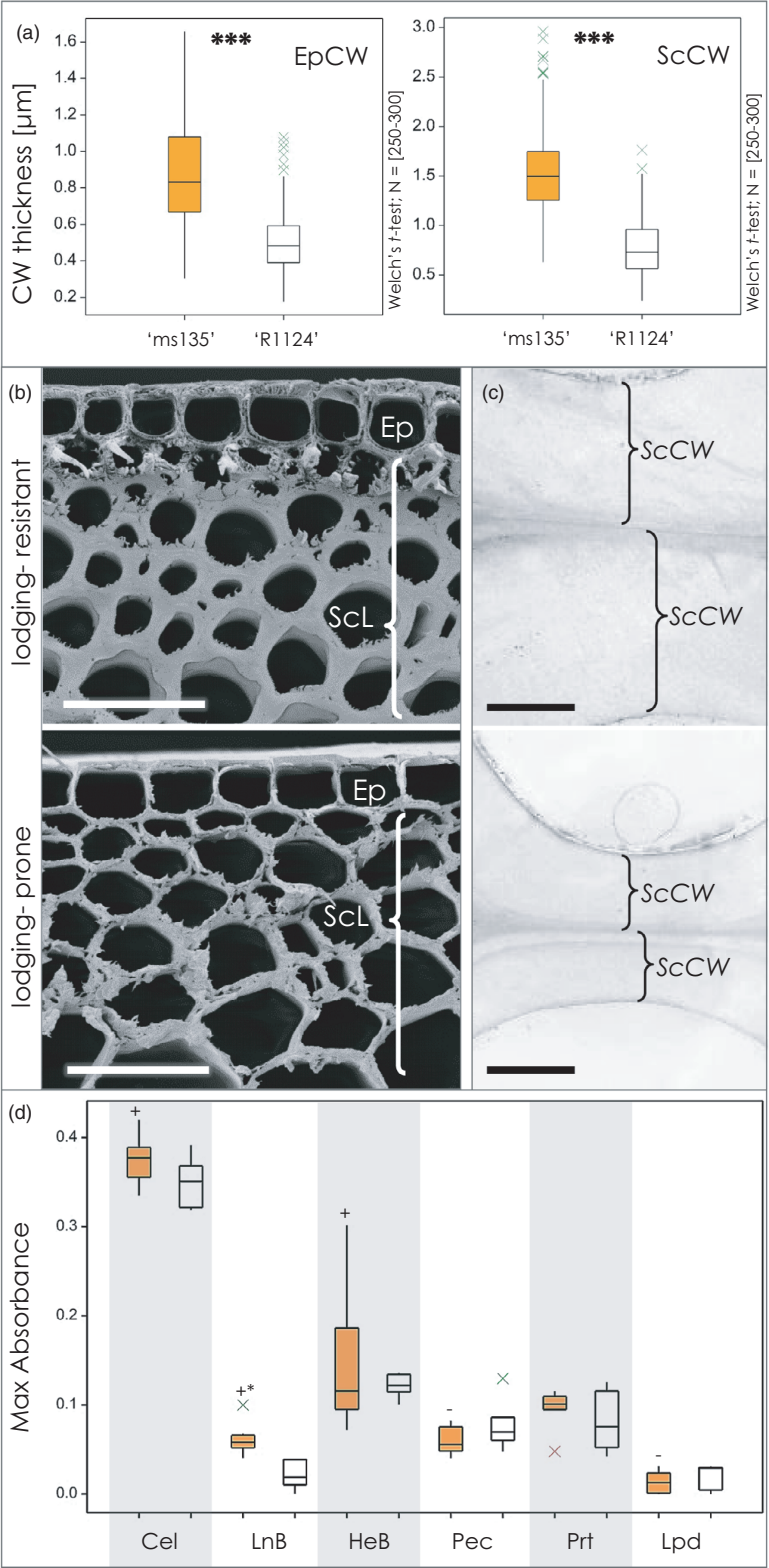
Cell wall structure and composition of the lodging‐resistant (‘ms135’) and lodging‐prone (‘R1124’) lines at the second basal internode. (a) Thickness of inner periclinal cell wall of epidermis (EpCW) and sclerenchymal cell wall (ScCW). (b) SEM image showing epidermis and sclerenchymal cell walls. Bar = 30 μm. (c) High resolution images in TEM showing two neighbouring sclerenchymal cell walls (ScCW) in lodging‐resistant and lodging–prone lines (upper and lower microphotograph, respectively). Bar = 1 μm. (d) The bar plot shows major cell wall‐related spectral compounds of lodging‐resistant (‘ms135’; mustard bars) and ‐prone line (‘R1124’, white bars). Abbreviations: Ep – epidermis, ScL – sclerenchyma tissue layer, ScCW – sclerenchymal cell wall, Cel – cellulose, LnB – lignin bulk, HeB – hemicellulose bulk, Pec – pectins, Prt – proteins, Lpd – lipids. Welch’s *t*‐test; *P < 0.05; **P < 0.01; ***P < 0.001.

The CW structure within the sclerenchyma and epidermis displayed variations between the two genotypes (Figure 4a–c). The sclerenchyma CW (ScCW) of the resistant plants was robust and almost twice as thick as that in the prone plants. Furthermore, the ratio of the sclerenchyma thickness to the diameter of the internode (ScR) was higher in the lodging‐resistant line (Table S1). The inner periclinal CW of the epidermis (EpCW) was also thicker in the resistant line as compared to the lodging‐prone line (0.87 ± 0.26 μm vs. 0.50 ± 0.16 μm, respectively; Figure 4a,b, Table S1).

Evaluation of these structural parameters [IVB, OVB, culm wall thickness (CWT), ScL, ScR, ScCW and EpCW] in F_2_ revealed that the characteristic traits of the lodging‐resistant parental line were fixed in their progeny (anatomy of the basal internode, Table S1).

Altogether, the results demonstrate that, even though the culm of the lodging‐resistant plants has thinner walls than its lodging‐prone equivalent, they are fortified by a higher proportion of sclerenchymal tissue and thicker CWs.

### Cell wall composition of lodging‐resistant rye internodes reveals chemotype shifts

**Figure 5:**
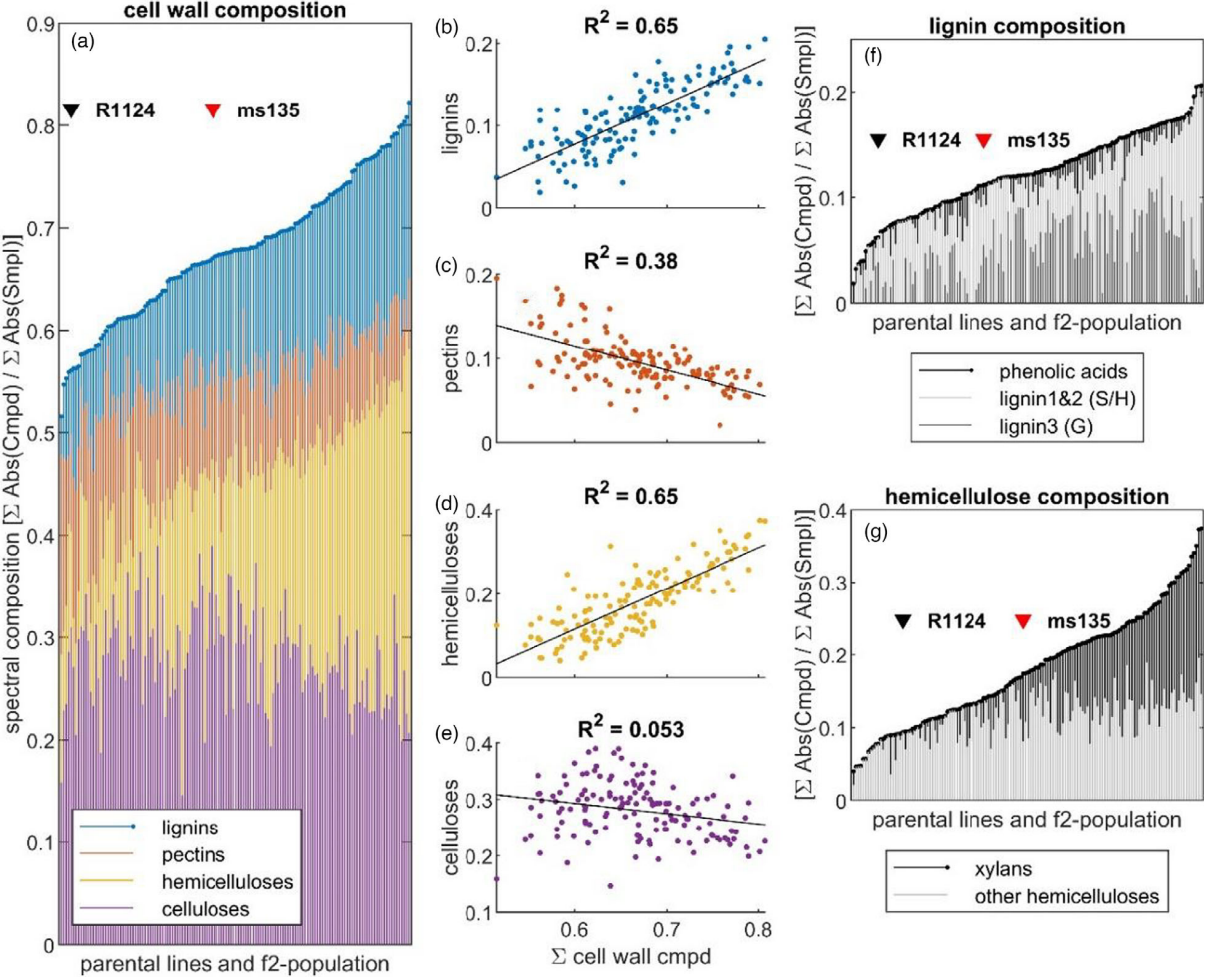
Compositional analysis of cell walls (CW) in the parental lines (lodging‐resistant ‘ms135’ and lodging‐prone ‘R1124’) and segregating F_2_ population of rye. (a) Comparative FTIR analysis of CW in parental lines and F_2_ population. The bar plot shows major and cell wall related spectral compounds of lodging‐resistant ‘ms135’ (EpI, red arrowhead) and prone ‘R1124’ (black arrowhead) line in respect to F_2_ population. (b–e) Pearson correlations between cell wall absorbance and its components: lignin (b), pectins (c), hemicelluloses (d) and cellulose (e). (f) Lignin signal further split into guaiacyl‐rich (Lignin3), syringyl/hydroxyphenyl‐rich (Lignin1&2) lignin species, as well as free aromatic acids. (g) Impact of xylans as a contributor towards high hemicellulose signals.

**Figure 6:**
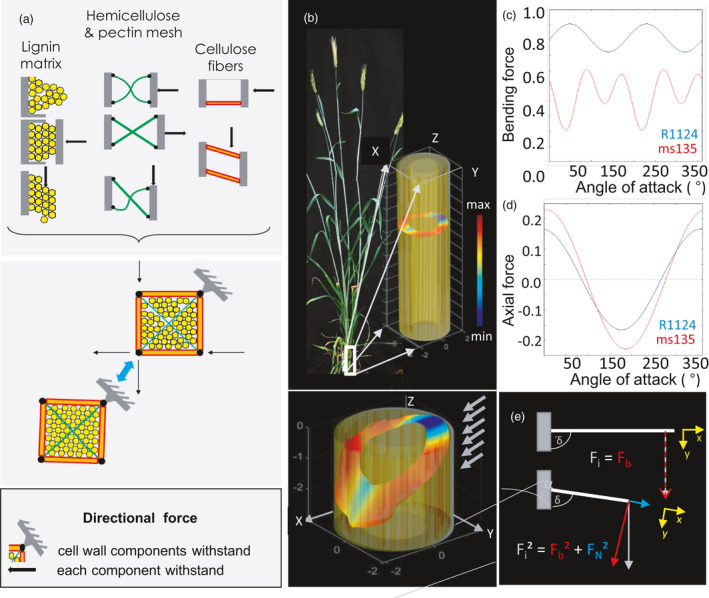
Role of stem components in mechanical robustness under stress. (a) Subcellular level scheme shows distinct cell wall components, which respond to directional forces by deformation (upper panel). When aligned together, the system acquires the ability to keep its shape (lower panel). This forms the basis for tissue specific mechanical properties. (b) Model of basal internode showing numerous individual fibres within the stem matrix (upper panel). Under stress (lower panel), the fibres distribute the impacting forces (arrows) out of the attacking plane, leading the deformation away from the main direction of attack (α). This allows for higher mechanical resistance towards bending. Absolute pressure on tissue is colour‐coded: minimal (blue) and maximal mechanical stress (red). (c) Interaction of bending and impacting forces computed for vascular bundles of stem during its response to the various directions of attack (e.g. wind direction) within the lodging‐resistant (ms138) and lodging‐prone (R1124) genotype. Estimation of *F*_b, α_ (*F*_impact_) is given in (*N*_b,,α_/*N*_impact,α_). (d) Impact of the vascular fibre orientation on stretching (axial forces). Estimation of *F*_N, α_ (*F*_impact_) is given in (*N*_N,α_/*N*_impact,α_). (e) Simplified model of a vascular bundle/fibre as a mechanical beam. Impacting Forces F_I_ can be translated into axial forces along the main axes of the fibre (*x,y*). The effective bending force *F*_b_ is the part of *F*_I_ which aligns with the *y*‐axis depending on the orientation (incline of the fibre, δ).

FTIR spectroscopy was applied to analyse the spectral fingerprints of the CW components of the two plant lines and their F_2_ segregating population. In the lodging‐resistant plants ‘ms135’, the signals detected for hemicelluloses, cellulose and lignin were higher than those from the lodging‐prone ‘R1123’ plants (Figure 4d). The results were further analysed using Cohen’s *D* effect size (estimating the magnitude of difference), which showed that the spectral variation of the resistant ‘ms135’ versus prone ‘R1124’ chemotypes favoured cellulose (*D* = 0.98), hemicelluloses (*D* = 0.69, main variation being xylans) and lignin species but demonstrated the opposite effect for pectins (*D* = 0.66). The FTIR spectral fingerprints of both parental lines and the F_2_ population CW components were also evaluated (Figure 5). While the variance within the progeny population was greater than between the parental lines, the extracted spectral CW characteristics explained 50%–80% of spectral features of all analysed plants. High variation was observed in the spectra of CW‐related characteristics, with the lodging‐prone parent at the lower end, and the lodging‐resistant parent exhibiting stronger signals (Figure 5a,f,g). A systematic change was observed in the composition of both hemicelluloses (Figure 5d) and in lignin (Figure 5b) signals within the cell wall‐related features. The former was primarily driven by increased xylan. In the lignin composition analysis, changes were evident in the ratio of syringyl‐ (LnS) and guaiacyl‐rich lignin species (LnG) (Figure 5f), both of which exhibited a higher spectral contribution in ‘ms135’ versus ‘R1124’ (*D* = 3.03, one‐way ANOVA: *P* = 0.00041), while shifting their ratio towards syringyl‐rich species. A minor negative trend was observed for pectins (Figure 5c), while no correlation was detected between cellulose and CW absorbance (Figure 5e). A xylan fingerprint was not detectable in 20% individuals of the 304/1 F_2_ population, but a medium statistical effect was observed for ‘ms135’ over ‘R1124’ (*D* = 0.68, *P* < 0.05). Mechanically, each of the cell wall components respond to directional forces in a different kind, but when aligned together, the composite acquires the ability to keep a defined structure much more rigid (Figure 6a).

In conclusion, our FTIR data give evidence to a significant increase in lignins and xylans in the CW as a chemotype characteristic of lodging resistance.

### High zinc and silicon levels are associated with lodging resistance

We characterized the elemental composition of the second basal internodes by quantifying 14 micro‐ and macro‐elements using ICP‐OES (Table S1). The lodging‐resistant line contained more than twice the amount of zinc than the lodging‐prone line and almost 50% more silicon. The data for the lodging‐resistant line further revealed lower levels of copper, calcium, potassium, magnesium, manganese and sulphur. The contents by dry weight of boron, iron, molybdenum, nickel, phosphorus and sodium were similar and highly variable in the two plant lines. In the present study, we provide the detailed elemental composition of lodging‐resistant and prone parental lines and their progeny (Table S1).

### Mechanical stress modelling

To investigate the role of the structural elements in mechanical robustness of stem, we further applied computational modelling of stress in two rye lines (‘ms135’ vs. ‘R1124’). The exact position of vascular bundles in the stem matrix was identified using MRI. Three‐dimensional data of segmented vascular structure were used to calculate and visualize the redistribution of external mechanical forces within the internal structure of internode (for details, see Experimental procedures). A simplistic model of the distortion occurring during wind attack is shown in a stem cross‐section of the lodging‐resistant plant ‘ms135’ (Figure 6b). In both lines, all fibres are involved, but cumulative action resulted in distinct response on stress in lodging‐resistant versus lodging‐prone line. The comparative analysis (Figure 6c,d) showed what exactly happened in the stems in terms of bending force and axial force (compressing/stretching force) under different directions of wind. The warping of the stem cross‐section out of the plane and the ripple‐like deformations occurred at the vascular bundle locations. This happened due to orthogonal stress deflection into the main axis of orientation of the vascular fibres. The lodging‐resistant line redistributes an attacking force on more elements (vascular bundles) as compared to the lodging‐prone line. Thus, the stalks better distribute pressure, allowing to avoid an excessive bending and to reduce damage under wind.

### Comparative analysis of wheat

Selected traits associated with lodging resistance in rye were also used for comparative analysis of wheat. For this purpose, we selected the lodging‐resistant line BW38990 (cv ‘Babax’/‘Baviacora’) and the lodging‐prone line BW38988 (cv ‘Pastor’). The lodging resistance of these lines has earlier been determined. Notably, both cultivars are semi‐dwarf and have the same plant height.

**Figure 7:**
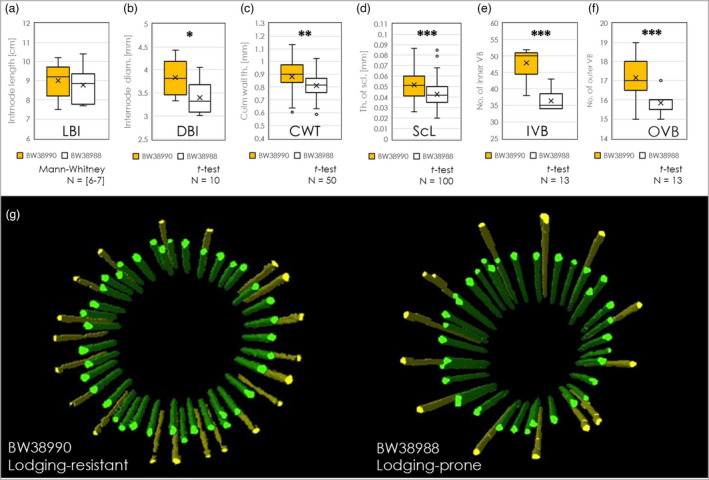
Comparative analysis of lodging‐resistant (BW38990—yellow bars) and lodging‐prone (BW38988—white bars) wheat lines. Lodging‐resistant (BW38990) and lodging‐prone (BW38988) wheat lines are characterized by similar length of the second basal internode (LBI, a). Nevertheless lodging‐resistant line’s internodes are significantly thicker than lodging‐prone internodes (DBI, b). Moreover, they are fortified with significantly thicker culm wall (CWT, c) and sclerenchymal layer (ScL, d), as well a higher number of inner (IVB, e) and outer vascular bundles (OVB, f), as it was observed in the ‘Stabilstroh’ rye line. The model of individual vascular bundles in inner (green) and outer vascular ring (yellow) in BW38990 and BW38988 based on 3D MRI data sets (g). Mann‐Whitney or *t*‐test; *P < 0.05, **P < 0.01, ***P < 0.001.

Our analysis revealed for both lines a similar length of the second basal internode (Figure 7a). The lodging‐resistant line’s internodes were significantly thicker than lodging‐prone internodes (Figure 6b). Similarly to the findings on rye (´Stabilstroh’ line), we found that the lodging‐resistant wheat line was characterized by a significantly thicker sclerenchymal layer (Figure 7d), but in contrast with the rye mutant, it had a thicker culm wall (Figure 7c). Similar as in ´Stabilstroh’ (rye) the number of vascular bundles was higher (Figure 7e,f).

The same MRI approach as used for rye was also applied to wheat. The models in Figure 7g show the distribution and number of vascular bundles in the intact second internode. The architecture appears similar in lodging‐resistant versus lodging‐prone plants, but the first one has a higher number of bundles. Thus, lodging resistance in both wheat and rye is associated with similar traits.

### QTL mapping of the traits of lodging resistance in rye

High‐throughput DArTseq genotyping has been used for efficiently profiling the whole genome of *Secale cereale* L. and targeting the gene‐rich regions of the genome (Bolibok‐Brągoszewska *et al*., 2009; Gawroński *et al*., 2016; Jaccoud *et al*., 2001; Milczarski *et al*., 2011, 2016). Building on this knowledge, we used the DArTseq approach to analyse the 304/1 F_2_ population.

**Figure 8:**
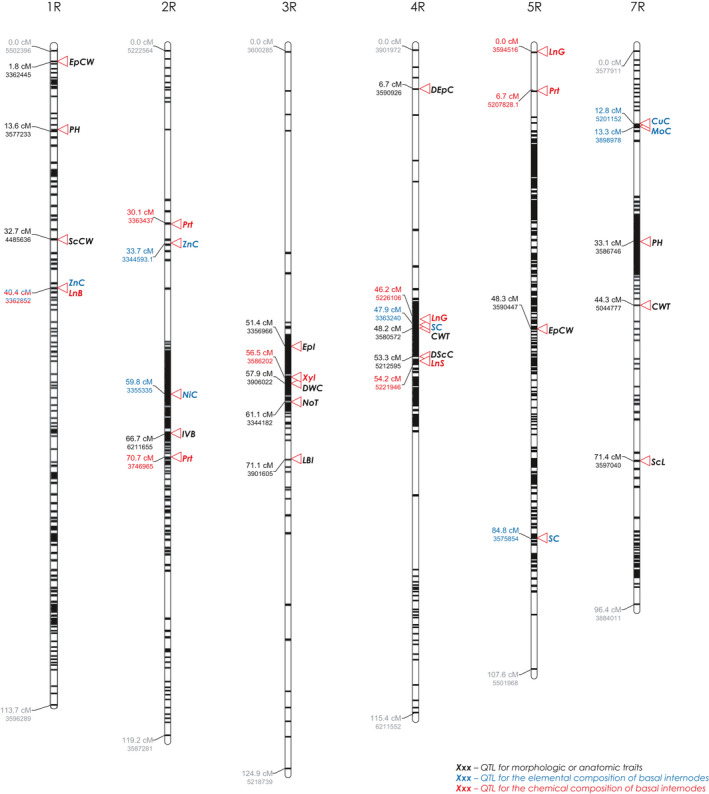
All the QTL associated with traits influencing lodging resistance found in 304/1 F_2_ population. The arrowheads show the position (numbers above the arrowheads) of maximum LOD (Logarithm of ODds) score. Abbreviations: CuC – copper content; CWT – culm wall thickness; DEpC – diameter of epidermal cell; DScC – diameter of sclerenchymal cell; DWC – dry weight of culms; EpCW – thickness of inner periclinal cell wall of epidermis; EpI – number of epidermal invaginations; IVB – number of inner vascular bundles; LBI – length of the second basal internode; LnB – lignin bulk; LnG – guaiacyl‐rich lignin; LnS – syringyl‐rich lignin; MoC‐ molybdenum content; NiC – nickel content; NoT – Number of tillers; PH – plant height; Prt – protein; SC – sulphur content; ScL – thickness of sclerenchyma; ScCW – thickness of sclerenchymal ce–l wall; Xyl ‐ xylan; ZnC – zinc content.

We constructed seven linkage maps based on 1041 segregating and co‐dominant DArTseq and SSR markers (Figure S1), covering 783.8 cM, with an average distance between two markers of 0.75 cM. Among the mapped DArTseq markers, 279 corresponded to the positions reported by Milczarski *et al*. (2016) for rye, and the remaining 762 were mapped on rye chromosomes for the first time. QTLs for the traits analysed in the present study were detected on all chromosomes but chromosome 6R (Figure 8; Table S2). For PH, two QTLs were detected in the F_2_ population: *QPh.ipk‐1R* and *QPh.ipk‐7R*, accounting for 14.1% and 16.5% of the observed phenotypic variance, respectively. Interestingly, mapping revealed only one QTL associated with the LBI, explaining 9.1% of the observed phenotypic variance. This was located on chromosome 3R at position 71.1cM and did not overlap with any PH QTLs. However, it was found to co‐localize with a QTL for DWC (explaining 30.1% of phenotypic variance for that trait) and NoT (explaining 31.9% of phenotypic variance). QTLs for EpI and xylan (Xyl) were also located on chromosome 3R, at 51.4 and 56.5 cM, respectively. Notably, the QTL for DWC was closely linked with a QTL for Xyl. The QTLs mapped for the diameter of sclerenchymal and epidermal cells as well as those for CWT (*QCwt.ipk‐4R*), sulphur content (*QSc.ipk‐4R*), LnG and LnS, were found to be co‐localized, but they did not overlap with the QTLs for ScCW (on 1R), EpCW (on 1R and 5R) or bulk cell wall (CWB; on 1R). Furthermore, the QTLs for PH, EpCW and ScCW on chromosome 1R co‐localized with one QTL of the two detected for zinc content (*QZnc.ipk‐1R*) and that for bulk lignin. The second QTL for zinc content was mapped on chromosome 2R and was co‐localized with that for IVB and nickel content, and two QTLs for proteins (Prt). The third QTL for Prt was localized on chromosome 5R together with QTLs for LnG, EpCW and sulphur content (SC).

The QTL for the trait considered critical from a mechanical point of view, the thickness of the sclerenchyma (ScL), was located on chromosome 7R and interestingly, was co‐localized with a QTL for CWT (*QCwt.ipk‐7R*), as well as those for copper and molybdenum content. This implies a complex genetic architecture from a mechanical aspect.

## Discussion

Our comparative analysis of resistant versus prone lines of rye and wheat plants revealed three main features, which could be of high relevance for breeding of mechanically resilient cultivars with high biomass production. First, the higher number of vascular bundles fortified the culm of the lodging‐resistant, as compared to the prone plant. Second, the cells of the sclerenchyma and epidermis of resistant culms exhibited characteristic thickening of cell walls. Third, chemical composition of cell walls shifted to higher levels of lignin and xylan, and tissues contained more zinc and silicon in the resistant versus lodging‐prone plants. The mathematical modelling approach showed how stem architecture of resistant plants allowed the distribution of mechanical stress. Distinct combination of traits that sustain the culm integrity under mechanical stress are heritable, as exemplified here for rye. The observed phenotypic and genotypic characteristics combined with the marker‐assisted selection approach using KASP markers (Tables S3 and S4, Figure S2) developed in this project present new possibilities for identifying and breeding genotypes with high biomass production and improved lodging resistance.

### Interaction of anatomy, microstructure, and composition—a mechanistic view

Mechanical robustness is related to the force distribution within load‐bearing structures of the plant and their mechanical material traits, which are also based on their chemical composition. In both rye and wheat, we demonstrated that lodging resistance goes along with more numerous vascular bundles, compared with the lodging‐prone lines (Figures 2 and 7), which agrees with previous observations (Khanna, 1991; Packa *et al*., 2015; Wang *et al*., 2006; Żebrowski, 1992; Zuber *et al*., 1999). For rye, we could further demonstrate that the degree of incline of the vascular bundles was increased in the lodging‐resistant line. This allows the culm to be more rigid due to an increase of mechanically stabilizing structures, for example, presenting a tighter formation of load‐bearing vascular bundles and stiffening indents within the surface architecture. The vasculature is embedded in a matrix of stem tissues with thickened CWs (Figure 4). The mechanical properties of the CW are determined not only by its thickness but also by the composition and dynamic interactions among its components (Simmons *et al*., 2016).

The structure and composition of the CW of ‘Stabilstroh’ includes the long, compact cellulose fibrils, which are perfectly suited for resisting forces directed along their orientation. The fibres are embedded in a mesh‐like matrix of hemicelluloses and pectins that due to the structure of their branching side chains acting like elastic, resisting any pulling forces. Finally, elasticity and strength are complemented by stiffness due to a lignin matrix that fills the gaps between the embedded holocellulose structures. In wheat, higher content of both lignin and cellulose was found to be associated with lodging resistance (Khobra *et al*., 2019). The balance of elasticity, strength and stiffness is of great importance. According to our analysis, the elevated xylan signals were associated with the lodging‐resistant phenotype, and a shifted composition of the complex lignin matrix in the lodging‐resistant line was evident (Figure 5a,b,f; Table S1). A similar effect has been observed in the maize *brittle stalk2 (bk2)* mutant, in which brittleness did not result from a loss of tensile strength, but from a decrease in the CW flexibility due to atypical lignin polymerization, caused by the lack of a proper cellulose matrix in the CW (Sindhu *et al*., 2007). Since monomers differ in the number of intramolecular polymerization sites and, therefore, the complexity of the lignin matrix, the role of these structural elements and their spatial arrangement is crucial for the mechanical robustness of the CWs. Overall, mechanical robustness is likely not due to increased stiffness (associated with lignin), but rather caused by a shift from hemicelluloses towards xylan, which adds elasticity and tensile strength to the CWs, leading eventually to the lodging resistance in ‘Stabilstroh’.

We have constructed a simplistic model of the distortion forces of a stem cross‐section during bending (Figure 6b), based on our MRI data sets (Figure 2b). The warping of the stem cross‐section out of the plane and the ripple‐like deformations occurred at the vascular bundle locations, due to orthogonal stress deflection into the main axis of orientation of the vascular fibres. If we consider an impacting force, for example, wind, the first impact is a result of the air pressure on the windward facing stem surface, which translates into a surface pressure on the outer stem layer. The stem tissues basically form ring‐like matrices of different densities and cell sizes, where cell size increases and density decreases towards the central axes of the stem. These radial patterns distribute pressure along their main axis, namely radial and axial in direction of the stem axis. With an orthogonal incident force this translates almost exclusively into radial pressure distributions resulting in bending and torsion. For simplification compositional changes, which effect cell size and density were neglected in our model computation due to insufficient information on related mechanical properties. The only main disruption towards that behaviour is presented by the vascular bundles, which, due to their angled orientation, redistribute a small portion out of plane along their fibre orientation upwards or downwards depending on the angle of attack towards the incoming pressure. This is demonstrated by the modelled plains warping characteristic (Figure 6b) and further by the average force redistribution towards axial stem orientation, which changes depending on the direction of the incoming force. Depending on the orientation the vascular fibres are predominantly either leaning towards (fibre compression) or away from (fibre stretching) the attacking direction. The lodging‐resistant genotype ms135 demonstrates a higher capacity for this behaviour as seen in the amplitude of Figure 6d. As a result, the effective bending strain on vascular fibres, which are a mechanically significant feature in stem architecture is reduced in contrast to the actual applied force (Figure 6c).

### Role of mineral composition in resistance to lodging

The lodging‐resistant plants contained more than twice the amount of zinc and almost 50% more silicon than the lodging‐prone ones (Table S1). Especially, the effect of silicon has gained much attention in crop breeding and research as deposits of Si reportedly serve to stiffen the stems of cereals (Berry *et al*., 2004; Dorairaj *et al*., 2017; Ma and Yamaji, 2006, 2008). Much less is known about the effect of zinc on lodging resistance. It was reported that an increased supply with zinc enhances the lignin content in wheat by 60% (Zaheri *et al*., 2015). This functional link seems relevant because lignin is generally associated with mechanical support, filling spaces in the cell wall, and increasing the mechanical strength of it. Our study also associated elevated zinc content with higher fraction of lignin. In addition, Zinc is essential for auxin production (Broadley *et al*., 2007) and the dimerization of cellulose synthase (Cosgrove, 2005; Kurek *et al*., 2002). The 1B/1R translocation in wheat leads to an increase in grain zinc levels (Monasterio and Graham, 2000), which is in line with the QTL, we mapped on chromosome 1R. Sadeghzadeh *et al*. (2015) identified four regions in the barley genome that were associated with the seed zinc content, located on the long arm of 3H, the short arm of 4H and the short and long arms of 2H. According to previous synteny analysis (Bauer *et al*., 2016; Martis *et al*., 2013), some of these positions may correspond to QTLs identified here (Figure 8, Table S2). In perennial wild rye, *Leymus cinereus* and *Leymus triticoides,* a QTL for zinc content on chromosome 1a corresponded to four barley metallothionine genes, whereas one on 2a corresponded to two yellow stripe‐like loci that include a heavy metal transporter gene affecting copper, iron, manganese and zinc accumulation (Curie *et al*., 2001; Yun *et al*., 2015). Considering the homology of the chromosomes of this allotetraploid *Leymus* species to those of barley and wheat, the QTL found in the present study may correspond to that described by Yun *et al*. (2015).

### Implications of structural and cell wall‐related traits for lodging resistance

Our results corroborate the idea that lodging resistance in rye relies on the efficient trade‐off between the culm structure and its composition. The culm of the lodging‐resistant ‘ms135’ mutant was strengthened with thicker‐walled sclerenchyma (Figure 4a–c; Table S1) and more vascular bundles, compared to the lodging‐prone ‘R1123’ line (Figure 2a–c; Table S1). Similar features were identified here also for lodging‐resistant wheat. We identified QTLs for each of these features on a high‐density genetic map (Figure 8; Table S2). Two QTLs for CWT were located on chromosomes 4R and 7R, a QTL for IVB on chromosome 2R, and a QTL for the thickness of the sclerenchymal tissue on chromosome 7R. The vasculature—in addition to its primary function circulating water and nutrients—contributes to the stiffness of the stem (Rüggeberg *et al*., 2009). In fact, the quantity and size of vascular bundles are correlated with lodging resistance in cereals (rye: Kubicka and Kubicki, 1981; wheat: Wang *et al*., 2006; Packa *et al*., 2015; maize: Sindhu *et al*., 2007).

The critical element determining the bending stress resistance of culm is the sclerenchyma tissue and its cellular components (rice: Matsuda *et al*., 1983; Ookawa *et al*., 2016; Ookawa and Ishihara, 1993), in which numerous vascular bundles were integrated. Thick‐walled, lignified sclerenchyma cells at the periphery of the stem are usually associated with mechanical strength and lodging resistance in various cereal crops (Aohara *et al*., 2009; Cosgrove, 2005; Kong *et al*., 2013). The picture around the interaction of traits became clearer with the study of the *brittle stem* (*bs*) mutation in rye, which resulted in fewer vascular bundles in the basal internodes, an aberrant structure and composition of the sclerenchyma tissue, and corresponding physicochemical alterations (Konovalov *et al*., 2013a; Kubicka and Kubicki, 1981). According to the present study on the progeny of the lodging‐resistant and prone lines, the QTL responsible for ScL thickness and one of the two QTLs for CWT co‐localized on chromosome 7R. Detailed ultrastructural analyses of culm tissues revealed significantly thicker sclerenchymal (ScCW) and epidermal (EpCW) CWs in the lodging‐resistant plants (Figure 4a–c, Table S1). Moreover, mapping analysis uncovered that a QTL for ScCW and one of the two QTLs for EpCW localized to the same chromosome (1R) but did not overlap with a QTL for the ScL thickness (7R).

Further study on CW composition and QTL mapping for lignin (LnB, LnG and LnS) could help to elucidate their functional relevance in the stem’s mechanical properties. For instance, the marker with the highest LOD (logarithm of the odds) score for ScCW (chromosome 1R) may correspond to the location of ferulate‐5‐hydroxylase, an enzyme involved in lignin synthesis (Andersen *et al*., 2008; Barrière *et al*., 2007; Boerjan *et al*., 2003; Ralph *et al*., 2004). The QTLs underlying the sclerenchymal and epidermal cell diameters co‐localized on chromosome 4R, but whether a single genetic mechanism regulates these traits remains unknown.

### Relevance of biomass‐related traits for breeding

Plant height is usually negatively correlated with basal internode breaking strength, suggesting that short varieties are more resistant to lodging (Börner and Korzun, 1998; Crook and Ennos, 1994; Miedaner *et al*., 2012; Sarker *et al*., 2007). Consequently, the *Rht* dwarfing alleles have been successfully used for breeding lodging‐resistant wheat. While dwarfism is unfavourable for biomass production, the search for alternative solutions continues, using innovative combinations of genome editing and synthetic biology, investigation of spontaneous mutants, genomic prediction studies, and other approaches (Fan *et al*., 2017; Jency *et al*., 2020; Juliana *et al*., 2019; Kashiwagi *et al*., 2008; Zhu *et al*., 2017). The ‘Stabilstroh’ mutant contains the desired combination of lodging resistance with an increased plant height, making it a prime candidate for producing lodging‐resistant rye varieties with tall posture and high biomass production. The present study revealed that *QPh.ipk‐1R* may correspond to the location of the recessive dwarfing gene *dw4* (Börner *et al*., 1996; Melz, 1989) or the dominant gene *Ddw3* (Stojałowski *et al*., 2015). The dominant dwarfing gene *Ddw2* (Börner *et al*., 1996) and the recessive genes *dw1*, *ct1* and *ct3*, all located on chromosome 7R, co‐locate with our *QPh.ipk‐7R* and correspond to the plant height QTL described by Milczarski (2008). Moreover, a QTL for the length of the second basal internode is located on the long arm of chromosome 3R, similar to the QTL described by Milczarski (2008).

The CWT and the width of mechanical tissue are two of the main parameters affecting the stem resistance to lodging (Kashiwagi *et al*., 2008; Kong *et al*., 2013; Konovalov *et al*., 2013b; Okuno *et al*., 2014; Wang *et al*., 2006; Zhang *et al*., 2017). Greater CWT enhances the mechanical strength of the stem and its endurance against lodging. In contrast to wheat (Figure 7c), the lodging‐resistant mutant used in our study was characterized by a smaller CWT than the lodging‐prone plants, testifying to the uniqueness of this genotype, and suggesting that other parameters, such as the thickness of sclerenchymal tissue, contribute more to its lodging resistance.

Tillering is crucial for biomass production and determination of grain yield (Donald, 1968; Hussien *et al*., 2014). This attribute is known to be associated with lodging in wheat (Tripathi *et al*., 2003), but not in rice (Kashiwagi *et al*., 2008). In the resistant rye plant, we identified only one major QTL (on chromosome 3R) that could be linked to the fivefold higher tiller production. Similarly, in barley, two QTLs mapped to chromosome 3H explained up to 50% of the observed phenotypic variation (Buck‐Sorlin, 2002). Mutations of genes that regulate NoT, for example, the *low number of tillers1* (*lnt1*) gene on chromosome 3H, can result in decreased tillering. Exceptions to this are the two genes *semi‐brachytic* (*uzu1*) and *semidwarf1/denso* (*sdw1/denso*) (Babb and Muehlbauer, 2003; Chono *et al*., 2003; Dabbert *et al*., 2009, 2010; Jia *et al*., 2011; Rossini *et al*., 2006). The former encodes a putative *brassinosteroid receptor* (HvBRI1), which is essential for tillering and stem elongation (Chono *et al*., 2003; Gruszka *et al*., 2011), and the latter also exerts a pleiotropic effect on tillering (Jia *et al*., 2011). Both are located on barley chromosome 3H, which is reciprocal to rye chromosome 3R. In contrast, rice genes *Dense and Erect Panicle (DEP1)* and *Ideal Plant Architecture1 (IPA1)/Wealthy Farmer’s Panicle (WFP)* also affect the vascular system (Huang *et al*., 2009; Jiao *et al*., 2010; Miura *et al*., 2010; Wang and Li, 2011), whereas such an overlap was not observed in rye, suggesting a distinct genetic background to rice.

## Experimental procedures

### Plant material

Plant material used in this study consisted of the winter rye (*Secale cereale* L.) parental lines ‘ms135’ (lodging‐resistant) and ‘R1124’ (lodging‐prone), as well as 129 individuals of a ‘304/1’ F_2_ population. The ‘ms135’ line, a spontaneous mutant characterized by stiff straw, tall posture, and lodging resistance (Flamme *et al*., 1997; experimental observations at Dieckmann GmbH, data unpublished), was discovered in partial population B (strain 6876) of ‘Kecskemeti törpe’, a Hungarian rye population (Melz and Dill, 1988). The wild type of the mutant is not known. Therefore, we used a normal rye ('R1124') for the cross enabling us to perform the genetic analysis. 'R1124' is a low tillering breeding line originated from the rye breeding company Dieckmann GmbH but known to be lodging susceptible. One single F1 seed was used to develop an F2 progeny comprising 129 single plants used for further analyses.

Individual plants were grown in pots (diameter: 14 cm; height: 12 cm) containing a 20% sand/80% compost soil mix (Substrate 2, Klasmann‐Deilmann GmbH, Geeste, Germany). For the first 8 weeks, the plants were vernalized in a growth chamber (4 °C, 12 h/12 h day/night regime) and hardened for 7 days to acclimatize gradually. Subsequently, the plants were grown in a greenhouse under controlled conditions (14 h/10 h, 16.5 ± 1.5/13.5 ± 1.5°C day/night regime at the early growth stage; 16 h/8 h, 21.5 ± 1.5/18.5 ± 1.5°C day/night until the maturity stage) in 60% humidity and 20 000 lx light intensity. In the vegetative growth stage, the plants were fertilized weekly with a 0.2% solution of Hakaphos Blau, replaced with 0.2% Hakaphos Rot solution (Compo Expert GmbH, Münster, Germany) during flowering and grain filling. The PH (including spikes) of the parental lines and the 304/1 F_2_ population were measured at harvest time. Additionally, 20 plants of two wheat cultivars: lodging‐resistant BW38990 (cv. ‘Baviacora’ (‘Babax’)) and lodging‐prone BW38988 (cv. ‘Pastor’) were used for comparative analysis of the key features. Wheat plants were grown in a compost soil/white peat/sand mix, fertilized weekly with Hekaphos Blau or Hekaphos Rot, under 16 h/8 h day/night regime with 17/15 °C temperature.

### SEM analysis

To characterize structural traits influencing lodging resistance, approximately 1 cm long transverse sections of the second basal internodes of straw of parents and 304/1 F_2_ plants were mounted on carbon‐coated aluminium specimen mounts. After gold‐coating using an Edwards S150B coater (Edwards High Vacuum Inc., Burgess Hill, UK) the sample surfaces were probed and imaged using a field‐emission SEM (Hitachi S‐4100, Hisco Europe, Germany) at an acceleration voltage of 10 kV. At least three cross‐sections were analysed for each plant (33 parental plants and 390 specimens from the F_2_ population).

### Light and transmission electron microscopy (TEM)

For comparative histological and ultrastructural analyses, transverse sections (˜1 mm thickness) of dry and fresh second basal internodes were used. Conventional and microwave‐assisted fixation, substitution and embedding were performed as defined in the protocol (Tables S5 and S6), with the exception that samples for LM were prepared without osmium tetraoxide post‐fixation. Samples were polymerized at 70 °C for 24 h in Spurr’s resin. Hardened sample blocks were trimmed with a Leica EM TRIM (Leica, Vienna, Austria) and cut into sections of 2‐μm and 70‐nm thickness for LM and TEM, respectively, with the aid of a Reichert‐Jung Ultracut S microtome (Leica). 1 μm thick sections were mounted on poly‐L‐lysine‐coated glass slides (Poly‐Prep, Sigma‐Aldrich, Darmstadt, Germany) and stained with 1% Azur II/1% methylene blue in 1% aqueous borax, while ultra‐thin (70 nm) sections were collected on 50 mesh hexagonal copper grids (Plano GmbH, Wetzlar, Germany) covered with polyvinyl formal (Sigma‐Aldrich Chemie GmbH, Germany). After contrasting with uranyl acetate and lead citrate (see Table S7) in a QG‐3100 Automated TEM Stainer (RMC Products, Boeckeler Instruments, Inc., Tucson, USA), the ultra‐thin sections were analysed using a FEI Tecnai™ G2 Sphera TEM (FEI Company, Eindhoven, the Netherlands) at 120 kV, and images were captured with an Olympus Veleta TEM CCD camera (Olympus GmbH, Hamburg, Germany). Staining with basic fuchsin was performed on semi‐thin sections to visualize lignified, suberized or cutinized CWs (Dharmawardhana *et al*., 1992; Kraus *et al*., 1998). Sections were analysed in a Zeiss Axio Imager M2 light microscope and imaged with a ZeissAxiocam MRc (Carl Zeiss GmbH, Jena, Germany).

To visualize lignin, 100 µm‐thick sections of fresh and dry second basal internodes were cut on a Leica VT1000S vibrating microtome (Leica Biosystems, Richmond, VA) and stained with phloroglucinol/HCl (McCarthy and Islam, 1999). Sections were analysed in brightfield using a Zeiss Axio Imager M2 light microscope. For processing and analysis of SEM, LM, and TEM images the Fiji open‐source platform for biological‐image analysis was used (Schindelin *et al*., 2012).

### Nuclear MRI and segmentation

The second basal internodes of the parental lines of rye, as well as two wheat lines, were analysed using a Bruker Avance III HD 400 NMR Spectrometer (Bruker BioSpin, Rheinstetten, Germany). A 3D data set (MSME sequence) was acquired using a 700 ms repetition time, a 6.6 ms echo time, a 4.2 × 4.2 × 13 mm field of view, and a matrix size of 168 x 168 x 26. Data processing was conducted using in‐house algorithms in MATLAB (R2018a, The MathWorks, Natick, MA). Segmentation and 3D reconstruction were performed using the Amira 2019.2 software (Thermo Fisher Scientific, Inc., Schwerte, Germany). The incline of the vascular bundles was calculated in MATLAB using the orthogonal plane as the plane of reference.

### Mechanical stress modelling

To visualize mechanical redistribution of external forces upon the segmented MRI stem structure, a 3D model was gridded into 1 mm thick cross‐sections along the main shoot axis (*z*‐axis). Each cross‐section was gridded again according to a radial grid in dα = 1‐degree steps around the main axis and radial increments dr = 1/20 (Ro–Ri) resulting in a 108000‐voxel gridded stem segment of 1.5 cm length. For each voxel the incoming mechanical force |Fin(α,*r*,*z*)| = |Fout(α,*r*,*z*)|. Fin is the sum of all impacting forces translated into the major directional axis of the voxel. These major axes follow the orientation of the grid according to α, r and *z*‐axis of the shoot geometry due to the radial pattern of different matrix tissues in the stem, while major axis shift towards the fibre orientation in voxels associated with vascular fibres (Figure 6e). The results were back‐transformed to a Cartesian coordinate system for the visualization. The model was tested for 360 different orthogonal attacking angles towards the stem to identify the optimal attack angle, which is depicted in Figure 6b. The 3‐dimensional tissue pressure distribution was modelled with a fictive force of 1 N/m² surface area. The model was integrated within the boundaries of *r* = *r*_max_:–*r*_max_, *z* = 0 : 15 mm and the starting condition of air pressure pin = [α −180°, 1 N/m², 0], which translates into Fin(α,*r*,*z*) = pin × projected voxel area(α,d*r*,d*z*). The bending characteristics were simplified to only reflect the pattern of force deflection within the stem structural elements rather than their composition specific traits, like Young’s module, which depend on the chemical composition within a specific voxel. To assess the sole effect of the vascular fibre orientation independently, the model was further simplified to compute axial (F_N_) and bending (F_b_) force for each fibre in relation with an impacting Force of 1 N per fibre, again depending on the angle of attack towards the main axis (α = 1‐360°). Axial (F_N_) and bending (F_b_) force partitioning changes according to the orientation of the fibres main axes towards the impacting force, which depends on fibre incline δ_fibre_ and position (α_fibre_, *r*_fibre_, *z*_fibre_). Variation over the Stem height was not considered in contrast to the previous model. For each NMR stem model, the average fibre bending and axial force was plotted over the angle of attack α in Figure 6b–d. The computation was done in MatLab (R2019b, The MathWorks).

### Analysis of elements

To evaluate silicon content, ˜25 mg dry ground material the second internode was used from each plant, according to a protocol adapted from Guntzer *et al*. (2010). Briefly, 8 mL 0.1 M Tiron solution (pH 10.5) was added to each sample, which was then placed in a shaking water bath for 1 h at 85 °C. The samples were then cooled and 7 mL 30% H_2_O_2_ was added to stop the Tiron reaction. The solution was shaken again at 85 °C until it became colourless. Before analysis, the samples were centrifuged, and their supernatants transferred into clean tubes. For the detection of other elements, ˜40 mg dry ground plant material was used. Samples were digested in nitric acid under pressure using a high‐performance microwave reactor (Ultraclave IV; MLS, Germany) and transferred to centrifuge tubes and diluted with deionized water. The detection of elements, including silicon, was performed using ICP‐OES (iCAP 6500 duo OES Analyzer with iTEVA Software, Thermo Fischer Scientific, Inc., Waltham, USA). An ASX‐520 Autosampler (ASXpress® plus system, CETAC Technologies, Omaha, USA) was coupled to the spectrometer for sample introduction. Yttrium was used as an internal standard.

### FTIR spectroscopy

Sample spectra of pulverized second basal internodes (˜2 mg) were produced from ATR‐FTIR measurements using a Tensor 27 FTIR spectrometer (Bruker Optics, Ettlingen, Germany) with a Globar light source under continuous purging with dry air. ATR absorbance spectra were recorded in the spectral range of 4000‐400 cm^−1^ at a spectral resolution of 6 cm. Each spectrum consisted of 64 co‐added scans. As a background reading, the spectrum of the empty ATR crystal was collected prior to measurement and subtracted automatically from each recorded spectrum using the OPUS software (Bruker Optics).

### FTIR data processing

OPUS files were imported into MatLab (R2018a, The MathWorks). Spectral data were reduced to a desired spectral range (wavenumber) of 1800–800 cm^−1^. Vector normalization of the data was carried out, and chemical components were detected using an adapted EMSC model (Bassan *et al*., 2012; Guendel *et al*., 2018), including the spectral features of holocelluloses and lignin by partial least squares regression. The principal component analysis was conducted with vector‐normalized and min‐max normalized spectral data of the parental lines and the F_2_ population. Principle component features, as well as CW‐related chemical components from available data sets, were used as FTIR traits for QTL mapping.

### Statistical analysis

Welch’s *t*‐test and Mann–Whitney test were used to compare traits between the parental lines. Statistically significant differences were identified, when *P* < 0.05. FTIR parameters were further investigated towards their effect size (Cohen, 1962 and Fritz *et al*., 2012) to support the significance claim of the statistical test. The following effect size thresholds were set: small effect: *D* > 0.2; medium effects: *D* > 0.5 and strong effects: *D* > 0.8. Pearson’s moment coefficient of skewness and kurtosis was applied to check the normality of data, using GenStat Sixteenth Edition (VSN International Ltd.). Pearson’s correlation coefficients were calculated to investigate the degree of linear dependence between traits, also using GenStat. Broad‐sense heritability (H^2^) was applied to estimate whether variation in a phenotypic trait in a population was due to genetic variation among individuals (Mahmud and Kramer, 1951).

### Genotyping and QTL mapping

DNA was isolated from leaves preserved in liquid nitrogen, according to Plaschke *et al*. (1995). 269 SSRs were amplified as described previously (Chebotar *et al*., 2003; Hackauf and Wehling, 2002; Khlestkina *et al*., 2004; Korzun *et al*., 2001; Röder *et al*., 1998; Saal and Wricke, 1999), and analysed for length polymorphism using an ALFwin Fragment Analyzer software package (Version 1.02; GE Healthcare Buchler GmbH, Braunschweig, Germany). 20 μL (50–100 ng/μL) extracted genomic DNA were used for genotyping, using the Rye GBS 1.0 platform (Diversity Arrays Technology Pty. Ltd.), as described previously (Bolibok‐Brągoszewska *et al*., 2009).

The genetic map was constructed in JoinMap 4.1 (Kyazma B.V.) based on the segregating markers (*χ^2^*‐test, *P* < 0.05) with <10% missing data. Non‐segregating markers and those of unknown parental origin were removed from the data set. The order of markers was determined by maximum likelihood mapping, and the distances between them were calculated by regression mapping and Kosambi’s mapping function (Kosambi, 1943). Groups were assigned to chromosomes using GenomeZipper (Martis *et al*., 2013), and graphical genotypes were inspected visually to verify the order of markers. Singleton data points were recorded as missing data in the data set and calculations were repeated.

Phenotypic information for the 304/1 F_2_ population and genotypic data based on 1041 markers were used for QTL analysis in GenStat Sixteenth Edition (VSN International Ltd, Hemel Hempstead, UK). To confirm the significance level with an LOD threshold of 3.0, a permutation test with 10 000 interactions for each trait was performed using QGene 4.3.10 (Joehanes and Nelson, 2008). Single‐marker regression analysis was performed initially, Simple Interval Mapping was carried out to obtain candidate QTL positions, which were used as cofactors in subsequent Composite Interval Mapping. The QSESTIMATE command was used to estimate QTL effects (Boer *et al*., 2014).

## Conflict of interest

The authors declare no conflicts of interest.

## Author contributions

AM, AB AG, MM and GM designed the study. AM, TR and MM investigated the ultrastructure of internodes. YTM analysed the element contents. EM, LB, AG and SO performed the MRI and modelling. AG performed FTIR spectroscopy experiments. GM, MR, AB, AG and TR interpreted the data. AM, HR and LB wrote the manuscript. AB edited the manuscript.

## Supporting information


**Figure S1** Seven linkage groups corresponding to rye chromosomes constructed in JoinMap basing on 1041 SNP and SSR markers.
**Figure S2** KASP assays for: 5215854 (A), 3353579 (B), 3596125 (C), 100074162 (D), 3349542 (E), and 5224120 (F).
**Figure S3** The lignified tissue and distribution of outer vascular bundles in lodging‐resistant (‘ms135’) and lodging‐prone (‘R1124’) line.
**Table S1** External morphology, anatomy of the basal internode, content of the elements, and cell wall components of parental lines (lodging‐resistant ‘ms135’ and lodging‐prone ‘R1124’) and 304/1 F_2_ population.
**Table S2** Summary of all the QTL found in 304/1 F_2_ population.
**Table S3** Sequences used for the development of KASP markers.
**Table S4** Genotyping by DArTseq and KASPs on 2 parental lines and 14 individuals from 304/1 F_2_ population.
**Table S5** Protocol of microwave‐assisted fixation, dehydration, and infiltration of basal internodes for LM.
**Table S6** Protocol of microwave‐assisted fixation, dehydration, and infiltration of basal internodes for TEM.
**Table S7** Uranyl acetate (UA) and lead citrate (PbC) staining program of ultra‐thin sections.
