# Supplementary material for: A mechanistic view on lodging resistance in rye and wheat: a multiscale comparative study
Source: Plant Biotechnol J. 2021 Sep 12;19(12):2646–61. doi: 10.1111/pbi.13689 (PMC8633492; doi:10.1111/pbi.13689)
Supplement: Supplementary file 1 — Figure S1 Seven linkage groups corresponding to rye chromosomes constructed in JoinMap basing on 1041 SNP and SSR markers. Figure S2 KASP assays for: 5215854 (A), 3353579 (B), 3596125 (C), 100074162 (D), 3349542 (E), and 5224120 (F). Figure S3 The lignified tissue and distribution of outer vascular bundles in lodging‐resistant (‘ms135’) and lodging‐prone (‘R1124’) line. Table S1 External morphology, anatomy of the basal internode, content of the elements, and cell wall components of parental lines (lodging‐resistant ‘ms135’ and lodging‐prone ‘R1124’) and 304/1 F2 population. Table S2 Summary of all the QTL found in 304/1 F2 population. Table S3 Sequences used for the development of KASP markers. Table S4 Genotyping by DArTseq and KASPs on 2 parental lines and 14 individuals from 304/1 F2 population. Table S5 Protocol of microwave‐assisted fixation, dehydration, and infiltration of basal internodes for LM. Table S6 Protocol of microwave‐assisted fixation, dehydration, and infiltration of basal internodes for TEM. Table S7 Uranyl acetate (UA) and lead citrate (PbC) staining program of ultra‐thin sections. [file PBI-19-2646-s001.pdf]

Tab.S1 External morphology, anatomy of the basal internode, content of the elements, and cell wall components of parental lines (lodging-resistant 'ms135' and lodging-prone 'R1124') and 304/1 F_2 population.

Abbreviations: heritability (H^2), mean value (μ), median (Q2), range ([Xmin,Xmax]), lower quartile (Q1), upper quartile (Q3), and standard deviation (SD). Statistical test: Welch *t*-test.

	Trait	P-value	H^2	Population	μ	Q_2	[Xmin,Xmax]	Q_1	Q_3	SD
External morphology	Plant Height PH [cm]	0.0491	0.8379	'ms135'	110.7	112.0	[102, 118]	103.0	117.0	6.9
				'R1124'	95.0	103.0	[70, 106]	85.8	105.2	15.2
				304/1	114.4	115.0	[85, 139]	108.5	121.0	10.0
	Length of the Basal Internode LBI [cm]	0.5397	0.2781	'ms135'	14.72	14.7	[9.8, 19.5]	13.20	17.40	2.90
				'R1124'	15.58	16.2	[12.6, 18.7]	13.42	17.20	2.44
				304/1	16.85	17.3	[12, 19.4]	15.70	18.42	1.86
	Number of Tillers NoT	0.0002	0.9751	'ms135'	16.0	16.5	[10, 21]	13.0	19.0	4.1
				'R1124'	3.0	2.0	[1, 7]	1.8	4.0	2.4
				304/1	8.32	8.0	[2, 20]	6.0	10.3	3.4
	Dry Weight of Culms DWC [g]	0.0007	0.9630	'ms135'	13.68	13.10	[6.6, 21.1]	11.10	17.10	4.98
				'R1124'	1.72	1.20	[0.2, 4.4]	0.58	2.68	1.65
				304/1	15.35	14.70	[4.9, 30.5]	10.70	19.00	5.76
	Dry Weight of a Single culm DWS [g]	0.0415	0.8496	'ms135'	0.840	0.834	[0.66, 1.11]	0.728	0.873	0.155
				'R1124'	0.546	0.629	[0.1, 0.7]	0.475	0.700	0.253
				304/1	1.944	1.893	[0.71, 3.78]	1.620	2.229	0.534
	Diameter of the Basal Internode DBI [mm]	0.0002	0.9435	'ms135'	3.638	3.673	[2.687, 4.685]	3.272	4.002	0.516
				'R1124'	4.381	4.339	[3.451, 5.617]	3.833	4.840	0.616
				304/1	4.266	4.244	[3.038, 5.630]	3.950	4.562	0.437
Anatomy of the basal internode	number of Epidermal Invaginations, EpI	0.0008	0.9330	'ms135'	5.8	4.0	[1, 15]	3.0	8.0	4.0
				'R1124'	1.2	2.0	[0, 3]	0.0	2.0	1.2
				304/1	4.9	2.6	[0, 31]	0.3	5.3	6.8
	Culm Wall Thickness CWT [µm]	< 0.0001	0.9732	'ms135'	341.8	323.9	[226.3, 503.0]	298.1	395.3	61.5
				'R1124'	389.7	388.8	[240.6, 512.4]	344.4	433.3	55.1
				304/1	466.5	469.9	[279.9, 643.5]	424.3	514.7	64.5
	Sclerenchymal Layer thickness ScL [µm]	< 0.0001	0.9967	'ms135'	70.83	67.85	[48.5, 113.7]	61.00	80.80	13.06
				'R1124'	49.59	48.60	[28.5, 78.9]	41.70	55.77	10.21
				304/1	72.19	72.03	[46.2, 101.7]	64.87	79.13	10.82
	Sclerenchyma to diameter Ratio ScR	< 0.0001	0.9987	'ms135'	0.0198	0.0189	[0.0141, 0.0297]	0.0173	0.0218	0.0035
				'R1124'	0.0112	0.0109	[0.0062, 0.0184]	0.0095	0.0125	0.0023
				304/1	0.0170	0.0167	[0.0109, 0.0237]	0.0151	0.0184	0.0027
	number of Inner Vascular Bundles, IVB	0.3234	0.5013	'ms135'	31.9	32.0	[27, 39]	30.0	33.0	3.0
				'R1124'	31.0	31.0	[29, 33]	30.0	32.0	1.4
				304/1	34.7	35.0	[24, 41]	32.7	37.0	3.2
	number of Outer Vascular Bundles, OVB	< 0.0001	0.9904	'ms135'	27.4	27.0	[24, 31]	25.3	29.0	2.1
				'R1124'	18.8	18.0	[15, 24]	16.5	21.5	2.8
				304/1	25.2	25.0	[13, 33]	23.0	27.7	3.9
	Diameter of Epidermal Cell DEpC [µm]	0.0684	0.7691	'ms135'	12.33	12.05	[8.82, 16.97]	10.68	13.74	2.09
				'R1124'	12.63	12.81	[8.08, 17.22]	10.95	14.33	2.29
				304/1	13.83	13.44	[9.96, 22.01]	12.53	14.54	2.23
	Diameter of Sclerenchymal Cell DScC [µm]	0.1928	0.6298	'ms135'	13.21	12.90	[9.05, 20.10]	11.06	15.08	2.61
				'R1124'	13.55	13.34	[8.63, 20.71]	11.24	15.58	2.93
				304/1	15.96	15.60	[9.30, 23.74]	14.53	17.12	2.41
	thickness of Sclerenchymal Cell Wall, ScCW [µm]	< 0.0001	0.9983	'ms135'	1.534	1.497	[0.630, 2.961]	1.256	1.748	0.420
				'R1124'	0.770	0.732	[0.239, 1.762]	0.563	0.96	0.283
				304/1	1.212	1.211	[0.604, 1.997]	0.975	1.408	0.289
	thickness of inner periclinal Cell Wall of Epidermis, EpCW[µm]	< 0.0001	0.9975	'ms135'	0.873	0.833	[0.304, 1.656]	0.668	1.079	0.259
				'R1124'	0.502	0.481	[0.178, 1.079]	0.390	0.593	0.162
				304/1	0.713	0.692	[0.396,1.153]	0.603	0.827	0.166
Analysis of the content of elements	Boron Content BC [µg/g]	0.3404	0.4889	'ms135'	13.15	12.65	[5.03, 23.42]	10.43	16.31	5.30
				'R1124'	16.15	13.88	[7.80, 34.32]	10.83	16.74	8.24
				304/1	7.81	6.51	[2.14, 24.41]	4.27	9.42	4.64
	Iron Content FeC [µg/g]	0.6625	0.1692	'ms135'	88.08	81.75	[62.28, 137.80]	64.89	100.00	28.16
				'R1124'	81.55	81.60	[58.24, 106.30]	72.50	89.85	17.18
				304/1	58.29	52.08	[23.91, 148.10]	39.86	71.32	24.45
	Molybdenum Content MoC [µg/g]	0.0628	0.8108	'ms135'	1.03	1.26	[0.44, 1.51]	0.54	1.47	0.50
				'R1124'	2.51	1.64	[0.86, 5.00]	1.28	4.65	1.83
				304/1	3.20	2.94	[0.34, 9.49]	1.63	4.29	2.07
	Nickel Content NiC [µg/g]	0.4814	0.3484	'ms135'	2.81	2.57	[1.54, 4.61]	2.02	3.36	1.04
				'R1124'	2.67	2.45	[1.99, 3.64]	2.17	3.31	0.67
				304/1	1.49	1.25	[0.42, 3.90]	0.93	1.78	0.78
	Phosphorus Content PC [µg/g]	0.0859	0.7931	'ms135'	2493	2446	[2036, 2864]	2308	2755	319
				'R1124'	3953	4331	[2076, 6060]	2429	5078	1637
				304/1	2748	2539	[879.3, 6403]	1909	3562	1250
	Sodium Content NaC [µg/g]	0.8148	0.0547	'ms135'	3142	3136	[2116, 4572]	2122	3770	956
				'R1124'	2923	2149	[1148, 6525]	1648	3920	2014
				304/1	4232	3854	[801.3, 9627]	2282	5836	2250
	Calcium Content CaC [µg/g]	< 0.0001	0.9775	'ms135'	4835	4365	[1365, 8708]	2918	7296	2460
				'R1124'	14686	12368	[11167, 24772]	12158	17415	4245
				304/1	6335	3617	[919.4, 24568]	1994	9240	6002
	Copper Content CuC [µg/g]	0.0026	0.9175	'ms135'	4.55	4.92	[1.92, 7.80]	2.18	6.17	2.29
				'R1124'	8.21	7.75	[2.58, 14.45]	5.82	9.71	3.41
				304/1	5.55	4.77	[2.02, 14.18]	3.76	6.90	2.53
	Potassium Content KC [µg/g]	0.0296	0.8525	'ms135'	56521	52575	[34106, 86250]	45390	66881	17694
				'R1124'	75388	76238	[50130, 92175]	66788	87375	14180
				304/1	58210	58057	[14492, 98035]	47865	66973	15978
	Magnesium Content MgC [µg/g]	0.0026	0.9302	'ms135'	1986	1740	[1550, 3124]	1611	2180	558
				'R1124'	3699	3842	[1546, 4993]	3163	4686	1132
				304/1	2829	2458	[719.7, 7597]	1322	3842	1764
	Manganese Content MnC [µg/g]	0.0022	0.9407	'ms135'	15.43	13.69	[8.69, 21.23]	11.68	19.91	4.91
				'R1124'	39.19	34.43	[27.17, 67.58]	28.68	42.88	14.97
				304/1	12.13	7.83	[3.18, 79.64]	5.65	12.52	12.96
	Sulphur Content SC [µg/g]	0.0005	0.9460	'ms135'	3805	3708	[3223, 4513]	3574	4102	448
				'R1124'	8294	8069	[6058, 11752]	6622	9195	2117
				304/1	5135	4540	[1568, 12141]	3176	6655	2668
	Zinc Content ZnC [µg/g]	0.0003	0.9649	'ms135'	116.80	104.50	[72.47, 160.70]	99.88	142.70	30.62
				'R1124'	46.07	46.88	[24.68, 60.45]	40.58	56.97	12.95
				304/1	94.06	86.00	[34.86, 246.70]	62.35	111.40	44.40
	Silicon Content SiC [µg/g]	0.0079	0.8936	'ms135'	6960	7238	[3866, 9639]	5489	8409	1805
				'R1124'	4903	3848	[3090, 7566]	3532	6953	1803
				304/1	5419	5387	[3989, 8443]	4795	5783	797
Cell wall content analysis by FTIR	Bulk Cell Wall CWB [max abs.]	0.1721	0.6838	'ms135'	0.2561	0.2463	[0.2104, 0.3138]	0.2292	0.3053	0.0415
				'R1124'	0.2316	0.2263	[0.2105, 0.2633]	0.2131	0.2275	0.0224
				304/1	0.3638	0.3493	[0.1863, 0.6257]	0.2710	0.4368	0.1053
	Bulk Lignin LnB [max abs.]	0.0004	0.9641	'ms135'	0.0656	0.0683	[0.0564, 0.0720]	0.0622	0.0719	0.0072
				'R1124'	0.0290	0.0315	[0.0143, 0.0388]	0.0243	0.0386	0.0119
				304/1	0.0820	0.0810	[0.0251, 0.1367]	0.0686	0.1000	0.0224
	Guaiacyl-rich Lignin LnG [max abs.]	0.0315	0.8620	'ms135'	0.0181	0.0184	[0.0068, 0.0285]	0.0098	0.0276	0.0102
				'R1124'	0.0055	1.09E-11	[4.25E-13, 0.0220]	5.26E-12	8.08E-11	0.0110
				304/1	0.0257	0.0259	[1.77E-16, 0.0655]	0.0054	0.0439	0.0198
	Syringyl-rich Lignin LnS [max abs.]	0.0139	0.8987	'ms135'	0.0373	0.0362	[0.0265, 0.0536]	0.0305	0.0475	0.0101
				'R1124'	0.0174	0.0151	[0.0006, 0.0388]	0.0136	0.0189	0.0159
				304/1	0.0462	0.0397	[6.18E-10, 0.1209]	0.0289	0.0601	0.0240
	Holocellulose Hol [max abs.]	0.6880	0.1460	'ms135'	0.1905	0.1778	[0.1540, 0.2448]	0.1648	0.2348	0.0368
				'R1124'	0.2026	0.1948	[0.1719, 0.2490]	0.1839	0.2032	0.0335
				304/1	0.2818	0.2725	[0.1388, 0.5194]	0.2050	0.3459	0.0901
	Cellulolse Cel [max abs.]	0.1414	0.7183	'ms135'	0.3825	0.3789	[0.3488, 0.4202]	0.3624	0.3862	0.0233
				'R1124'	0.3382	0.3368	[0.3186, 0.3605]	0.3225	0.3605	0.0208
				304/1	0.3367	0.3350	[0.1975, 0.4587]	0.3053	0.3696	0.0470
	Bulk Hemicellulose HeB [max abs.]	0.4454	0.3870	'ms135'	0.1261	0.1148	[0.0720, 0.1892]	0.1012	0.1832	0.0473
				'R1124'	0.1188	0.1206	[0.1007, 0.1334]	0.1193	0.1334	0.0136
				304/1	0.2105	0.1986	[0.0402, 0.4704]	0.1151	0.2861	0.1048
	Pectins Pec [max abs.]	0.2830	0.5628	'ms135'	0.0644	0.0654	[0.0477, 0.0821]	0.0491	0.0753	0.0149
				'R1124'	0.0838	0.0705	[0.0645, 0.1297]	0.0645	0.0712	0.0308
				304/1	0.0713	0.0691	[0.0024, 0.1581]	0.0572	0.0794	0.0244
	Xylan Xyl [max abs.]	0.2672	0.5799	'ms135'	0.0395	0.0246	[3.48E-12, 0.0944]	0.0153	0.0875	0.0388
				'R1124'	0.0250	0.0252	[7.24E-11, 0.0498]	0.0057	0.0446	0.0258
				304/1	0.0976	0.0796	[1.18E-15, 0.3276]	0.0095	0.1642	0.0898
	Proteins Prt [max abs.]	0.1821	0.6764	'ms135'	0.1039	0.1038	[0.0946, 0.1155]	0.0965	0.1096	0.0085
				'R1124'	0.0890	0.0939	[0.0423, 0.1256]	0.0557	0.1122	0.0376
				304/1	0.0757	0.0745	[4.45E-15, 0.1856]	0.0364	0.1085	0.0458
	Lipids Lpd [max abs.]	0.4667	0.3641	'ms135'	0.0132	0.0113	[6.51E-14, 0.0315]	0.0022	0.0218	0.0137
				'R1124'	0.0224	0.0292	[2.36E-10, 0.0312]	0.0061	0.0293	0.0150
				304/1	0.0103	0.0042	[1.13E-15, 0.0471]	9.89E-12	0.0188	0.0131

Tab.S2 Summary of all the QTL found in 304/1 F_2 population.

Abbreviations: Chr.- chromosome; LOD – Logarithm of Odds; % Expl.Var. – percent of phenotypic variance that can be explained by the QTL; Add./Dom. effect - additive/dominance effect; Dom. allele – dominant allele

PH – plant height; LBI – length of the second basal internode; NoT – number of tillers; DWC – dry weight of culms; EpI – number of epidermal invaginations; CWT – culm wall thickness; ScL – thickness of sclerenchymal layer; IVB – number of inner vascular bundles; DEC – diameter of epidermal cell; DSC – diameter of sclerenchymal cell; ScCW – thickness of sclerenchymal cell wall; EpCW – thickness of inner periclinal cell wall of epidermis; MoC - molybdenum content; NiC – nickel content; CuC – copper content; SC – sulphur content; ZnC – zinc content; LnB – lignin bulk; LnG – guaiacyl-rich lignin; LnS – syringyl-rich lignin; Xyl - xylan; Prt – protein.

Trait	QTL ID	IDLocus	Chr.	Position [cM]	LOD	% Expl. Var.	Add. effect	High value allele	Dom. effect	Dom. allele
PH	*QPh.ipk-1R*	3577233	1R	13.6	4.246	14.076	5.150	'ms135'	1.811	'ms135'
	QPh.ipk-7R	3586746	7R	33.1	5.688	16.522	5.649	'ms135'	1.486	'ms135'
LBI	*QLbi.ipk-3R*	3901605	3R	71.1	3.02	9.133	0.744	'R1124'	0.565	'R1124'
NoT	*QNot.ipk-3R*	3344182	3R	61.1	9.502	31.886	2.617	'ms135'	0.840	'R1124'
DWC	*QDwc.ipk-3R*	3906022	3R	57.9	9.551	30.118	4.513	'ms135'	1.078	'R1124'
EpI	*QEpi.ipk-3R*	3356966	3R	51.4	3.108	10.707	2.201	'ms135'	2.190	'R1124'
CWT	*QCwt.ipk-4R*	3580572	4R	48.2	3.001	10.351	29.366	'ms135'	0.726	'ms135'
	QCwt.ipk-7R	5044777	7R	44.3	4.072	14.892	35.198	'ms135'	3.994	'ms135'
ScL	*QScl.ipk-7R*	3597040	7R	71.4	3.365	1.618	1.818	'ms135'	7.008	'ms135'
IVB	*QIvb.ipk-2R*	6211655	2R	66.7	3.369	10.423	1.465	'ms135'	0.515	'ms135'
DEC	*QDec.ipk-4R*	3590926	4R	6.7	3.010	9.686	0.983	'ms135'	0.150	'R1124'
DSC	*QDsc.ipk-4R*	5212595	4R	53.3	3.641	1.682	0.442	'ms135'	1.533	'R1124'
ScCW	*QScw.ipk-1R*	4485636	1R	32.7	3.738	10.738	0.134	'ms135'	0.035	'R1124'
EpCW	*QEcw.ipk-1R*	3362445	1R	1.8	5.333	3.412	0.041	'ms135'	0.124	'R1124'
	QEcw.ipk-5R	3590447	5R	48.3	3.737	7.690	0.063	'ms135'	0.066	'ms135'
MoC	*QMoc.ipk-7R*	3898978	7R	13.3	4.754	13.301	1.265	'R1124'	0.817	'ms135'
NiC	*QNic.ipk-2R*	3355335	2R	59.8	3.050	0.010	0.011	'ms135'	0.490	'R1124'
CuC	*QCuc.ipk-7R*	5201152	7R	12.8	5.962	14.464	1.305	'R1124'	1.272	'ms135'
SC	*QSc.ipk-4R*	3363240	4R	47.9	3.160	0.723	390.102	'R1124'	1648.748	'R1124'
	QSc.ipk-5R	3575854	5R	84.8	3.000	0.627	303.571	'R1124'	1577.704	'R1124'
ZnC	*QZnc.ipk-1R*	3362852	1R	40.4	3.037	8.270	17.566	'ms135'	20.072	'ms135'
	QZnc.ipk-2R	3344593.1	2R	33.7	3.292	2.780	10.095	'R1124'	27.204	'ms135'
LnB	*QLnb.ipk-1R*	3362852	1R	40.4	3.174	10.700	0.00609	'ms135'	0.00148	'ms135'
LnG	*QLng.ipk-4R*	5226106	4R	46.2	3.884	7.222	0.00715	'ms135'	0.01054	'ms135'
	QLng.ipk-5R	3594516	5R	0.0	3.510	8.319	0.01019	'R1124'	0.00040	'R1124'
LnS	*QLns.ipk-4R*	5221946	4R	54.2	3.665	7.713	0.01011	'R1124'	0.00847	'R1124'
Xyl	*QXyl.ipk-3R*	3586202	3R	56.5	3.629	12.200	0.03225	'ms135'	0.01751	'ms135'
Prt	*QPrt.ipk-2R.1*	3363437	2R	30.1	4.310	6.297	0.02336	'ms135'	0.00034	'R1124'
	QPrt.ipk-2R.2	3746965	2R	70.1	3.220	2.586	0.01847	'ms135'	0.00909	'ms125'
	QPrt.ipk-5R	5207828.1	5R	6.7	3.170	0.131	0.01897	'ms135'	0.00128	'R1124'

Tab.S3 Sequences used for the development of KASP markers.

The sequences provided with DArT markers are highlighted with yellow colour.

SNP ID	Sequence
5218584	CGCGCTCCGACATGGACATGGGCTTCGCCGGCCCCACCACCTGCCCGCCGCCGCCGCCGTCCCCGCCTTCAG[T/C]AAGCGTCCCCTGCTGCATGATTGACGGCTGCAGGAGCTCATGGTTGTTGCAGCCGCCGTCGCCGGCGCCACCAC
3349542	CTTGTCGAGCCACCGGTATGTGAATGTGTTCGTGTGTGAGGGAGGCACGATGTTGATCTGGAGGGTGTCAAAGCATCATGGCCAAACCTCACGACCGTAAGTACAATGAGCTAGAATGTGAT[T/C]AATGTTGTCCTTGTCATGTAAGCATGTGAAGCAAGCAAAAGGCACATCCTGCAGATCGTGCCTAGCACGTCTGTCAGAGGTCCATCGTCGATATTG
5224120	CTGTAAATACATAACGATTTAATGGGTGCTGAACATACAAATCCCCATCCCTCCTTCGTGAACTCCTGACCGACTATTCTGAAGTTGCAGTGGGTTGACAGGAAGATCTCAACACATGGCATCTCGTCAACAACTGCAGCAACACC[G/A]CCGTCTTTCGGCCCACGATTAAGGGCATTGGCGTACTCCTCAACAGTGCTCAGTGGTACTAACCGGGATTCAGGT
3596125	GCCGGTTGCCTAAATTTCCAGCGCATGCATATGGCCACGTATGGTAGCCCGACCTGGTCAAACTAGATAAGGGAGAGCCCGAGTCACTTCGATCCCAAATCCA[T/C]AGCGGAGACCCCAGGGCCAGCCGCATCACCGACTGCAGGAAGAGCTCTAGGCGTGCTCGTCCTCGTCGGAAAAGCCCCTAGGAATGAGTGTGTAGGGTGTGATGCCGTTCGCGT
3353579	GAGCAGGCGCCGGTGACGGCGACGGGGCTGTTCTGCCGGCCGCTGAAGACGCTGGACCTCTTCCCCGGCGCGATCAAGGAGGAGCAGCGCGACGTCGCCTAGC[C/T]ACCTCCAACCTATAGCTAGCTGCAGCAGCAGTCGTCGTCGTCGTCGTAGTACGTAGAGCAGTCCGTACGTTACGCGCGCGCGCGTTGTACGTACGCAGGTAGAGCAGCAGCAG
100074162	CTCCACCTCGAGCGAGCAAACGACAGCCAGCGGAAGCAAGAGC[G/A]CCCAGCAGGCCTAGGTGCGCCGCCCAAGCTCTTCTTCACGACGGCGCCGAGCCGCCTGCGCGCGTGGCAACACGCTCCTCCGCCGGTGCTGCGTGAGCTGA

Tab.S4 Genotyping by DArTseq and KASPs on 2 parental lines and 14 individuals from 304/1 F_2 population.

ID SNP	Marker type	Line															
		ms135	R1124	304/1-1	304/1-2	304/1-13	304/1-18	304/1-21	304/1-22	304/1-25	304/1-28	304/1-37	304/1-38	304/1-47	304/1-48	304/1-68	304/1-100
5218584	DArT	0	1	0	0	1	2	0	2	2	2	0	1	1	2	2	2
	KASP	Y	C	T	T	C	Y	T	Y	Y	Y	T	C	C	Y	Y	Y
3353579	DArT	0	1	0	2	1	2	0	0	2	2	2	1	1	2	0	2
	KASP	T	C	T	Y	C	Y	T	T	Y	Y	Y	C	C	Y	T	-
3596125	DArT	1	0	2	1	1	2	0	2	0	2	2	2	0	2	1	0
	KASP	Y	Y	Y	Y	Y	Y	Y	Y	Y	Y	Y	Y	Y	-	Y	Y
100074162	DArT	1	0	2	2	2	0	1	2	2	2	1	0	2	2	1	0
	KASP	-	-	-	-	-	-	-	-	-	-	-	-	-	-	-	-
3349542	DArT	1	0	0	2	1	2	1	2	2	2	2	0	0	2	2	1
	KASP	C	Y	T	Y	-	Y	C	Y	Y	Y	Y	T	T	Y	Y	C
5224120	DArT	0	1	1	2	0	2	0	2	2	2	0	1	1	2	0	0
	KASP	G	A	A	R	G	R	G	R	R	R	R	A	A	R	G	G

Tab.S5 Protocol of microwave-assisted fixation, dehydration, and infiltration of basal internodes for LM.

Process	Chemical compound	Microwave irradiation	Time of irradiation	Vacuum
**Fixation**	2% GA + 2% FA in 50 mM cacodylate buffer (pH 7.2)	150 W	1 min.	**15 mm Hg**
		+ overnight incubation at RT		
**Washing**	1x<br>50 mM cacodylate buffer (pH 7.2)<br><br>2x<br>degassed ultrapure water	150 W	45 s	**-**
		+ 15 min incubation at RT		
**Dehydration**	Ethanol series:<br>30%, 40%, 50%, 60%, 70%, 80%, 90%<br>2x 100% EtOH<br><br>And 2x propylene oxide	150 W	45 s	**-**
		+ 15 min incubation at RT		
**Infiltration**	25% Spurr in PO<br>40% Spurr in PO	250 W	3 min	**5 mm Hg**
		+ 3 h incubation at RT		
	50% Spurr in PO	250 W	3 min	**5 mm Hg**
		+ overnight incubation at RT		
	60% Spurr in PO<br>70% Spurr in PO<br>80% Spurr in PO<br>90% Spurr in PO	250 W	3 min.	**5 mm Hg**
		+ 3 h incubation at RT		
	100% Spurr	250 W	3 min	**5 mm Hg**
		+ overnight incubation at RT		
	100% Spurr	250 W	3 min	**5 mm Hg**
		+ 3 h incubation at RT		

Tab.S6 Protocol of microwave-assisted fixation, dehydration, and infiltration of basal internodes for TEM.

Process	Chemical compound	Microwave irradiation	Time of irradiation	Vacuum
**Fixation**	2% GA + 2% FA in 50 mM cacodylate buffer (pH 7.2)	150 W	1 min	**15 mm Hg**
		+ overnight incubation at RT		
**Washing**	50 mM cacodylate buffer (pH 7.2)<br><br>2x<br>degassed ultrapure water	150 W	45 s	**-**
		+ 15 min incubation at RT		
**Post-fixation**	1% osmium tetroxide	-	2 min	**15 mm Hg**
		80 W	2 min	**15 mm Hg**
		-	2 min	**15 mm Hg**
		+ 1 h incubation at RT		
**Washing**	3x<br>degassed ultrapure water	150 W	45 s	**-**
		+ 15 min incubation at RT		
**Dehydration**	Ethanol series:<br>30%, 40%, 50%, 60%, 70%, 80%, 90%<br>2x 100% EtOH<br><br>And 2x propylene oxide	150 W	45 s	**-**
		+ 15 min incubation at RT		
**Infiltration**	25% Spurr in PO<br>40% Spurr in PO	250 W	3 min	**5 mm Hg**
		+ 3 h incubation at RT		
	50% Spurr in PO	250 W	3 min	**5 mm Hg**
		+ overnight incubation at RT		
	60% Spurr in PO<br>70% Spurr in PO<br>80% Spurr in PO<br>90% Spurr in PO	250 W	3 min	**5 mm Hg**
		+ 3 h incubation at RT		
	100% Spurr	250 W	3 min	**5 mm Hg**
		+ overnight incubation at RT		
	100% Spurr	250 W	3 min	**5 mm Hg**
		+ 3 h incubation at RT		

Tab.S7 Uranyl acetate (UA) and lead citrate (PbC) staining program of ultrathin sections.

Step No.	Process	Time
1	Filling the chamber with degassed Millipore water	1 min
2	Wait state (the staining chamber and tubes soak) to wet the grids	10 min
3	Filling the chamber with UA	24 s
4	Staining with UA	10 min
5	Disposing of UA (washing with degassed water)	2 min
6	Wait state (interim step before next command)	6 s
7	Filling the chamber with PbC	24 s
8	Staining with PbC	5 min
9	Disposing of PbC (washing with degassed water)	2 min
10	Wait state (interim step before next command)	6 s
11	Filling the chamber with degassed Millipore water	1 min

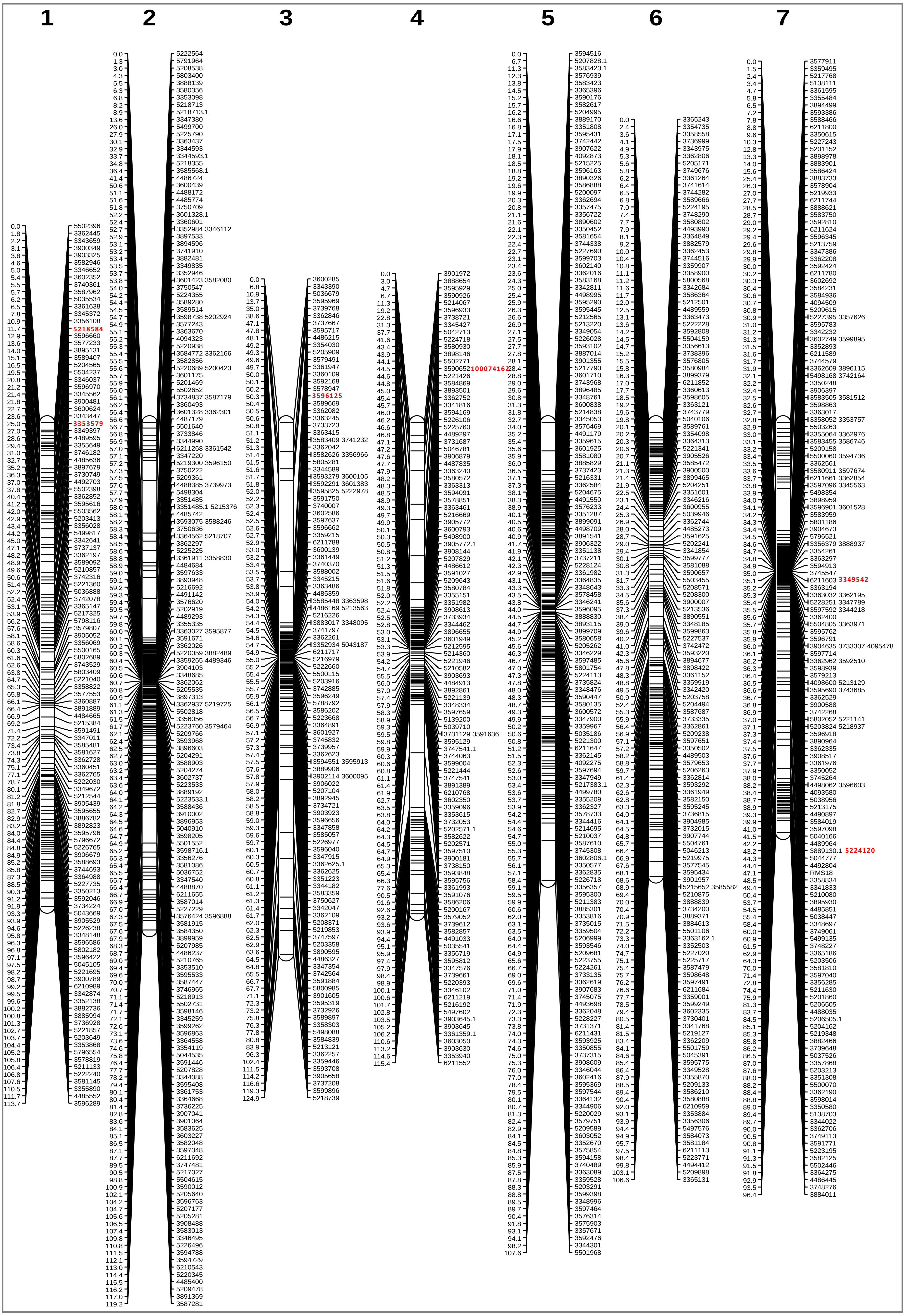

Fig.S1 Seven linkage groups corresponding to rye chromosomes constructed in JoinMap basing on 1041 SNP and SSR markers. SNPs used for the development of KASP markers marked with red font.

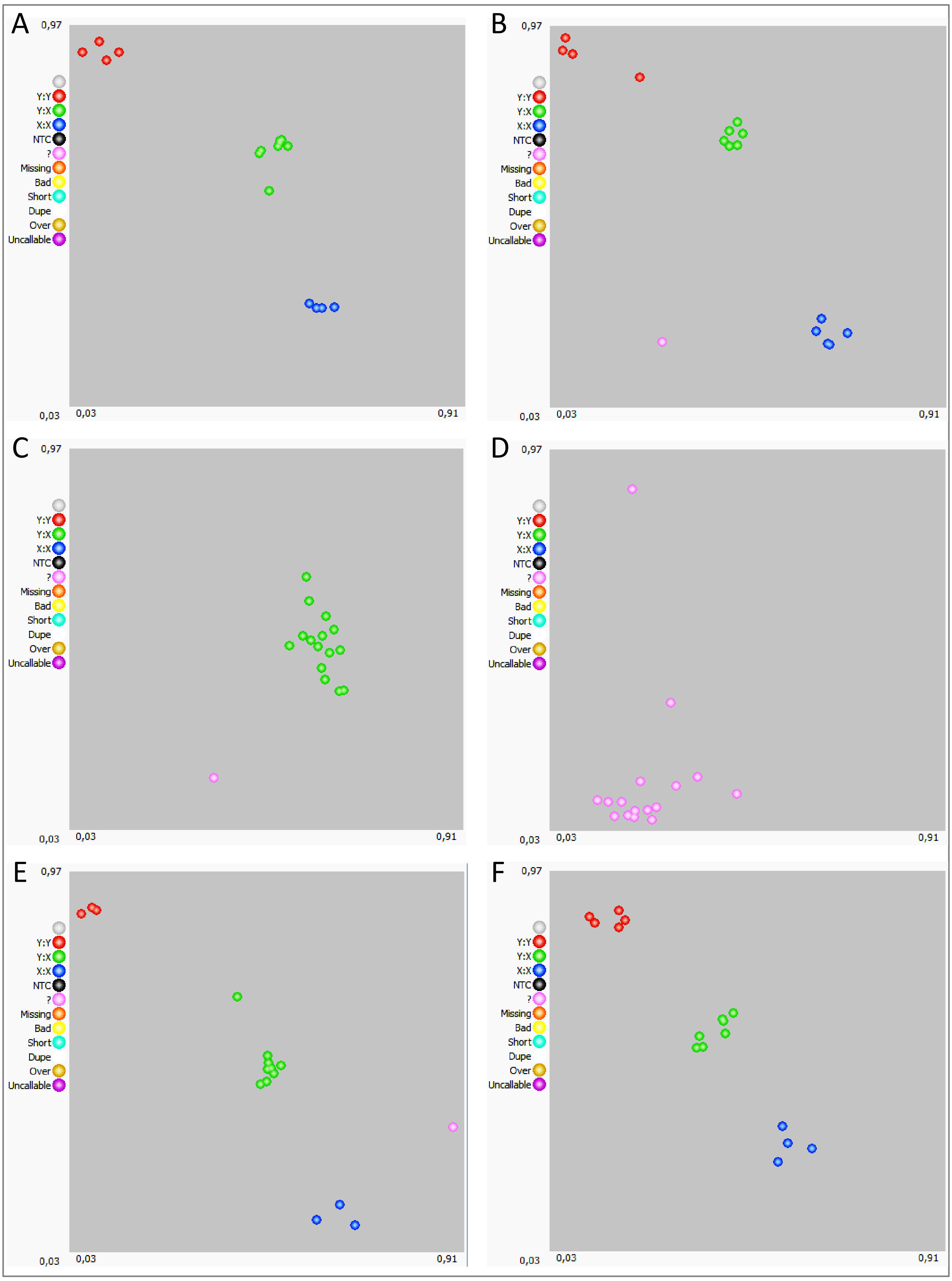

FIG.S2 KASP assays for: 5215854 (**A**), 3353579 (**B**), 3596125 (**C**), 100074162 (**D**), 3349542 (**E**), and 5224120 (**F**).

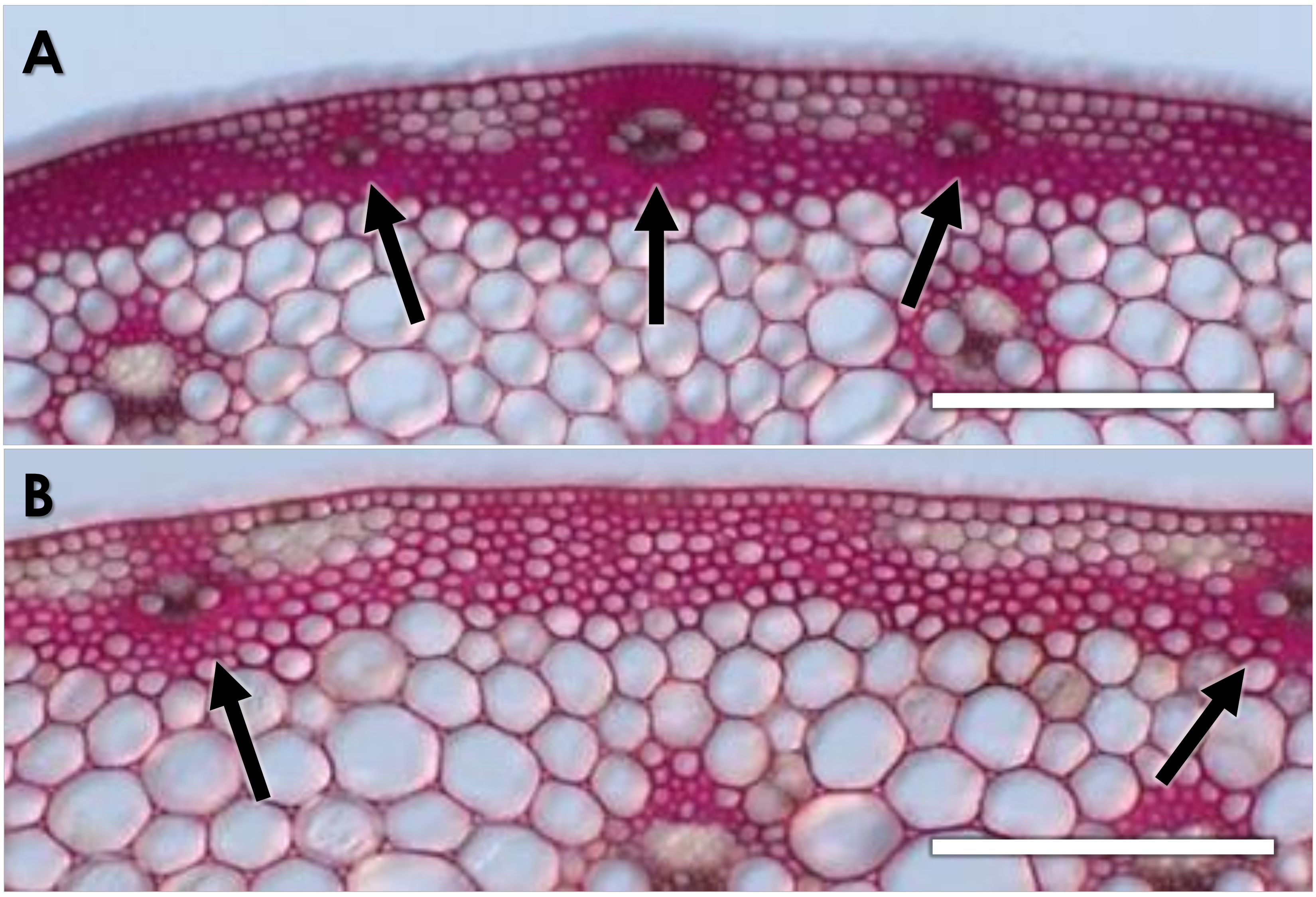

Fig.S3 The lignified tissue and distribution of outer vascular bundles in lodging-resistant ('ms135') and lodging-prone ('R1124') line.
(A-B) transverse sections in LM showing distribution of outer vascular bundles (OVB; black arrows) in the lodging-resistant and prone lines ('ms135' and 'R1124' respectively in A and B; phloroglucinol staining, DIC; bars = 200µm).
